# Comparative Analysis of Expert, Clinician, and Health Care User Interactions With Summary of Findings Tables: Usability Study

**DOI:** 10.2196/86045

**Published:** 2026-07-15

**Authors:** Nina Vitlov, Nensi Bralić, Tina Poklepović Peričić, Daniel Garcia-Costa, Emilia López-Iñesta, Elena Álvarez-García, Francisco Grimaldo, Ana Marušić

**Affiliations:** 1 Department of Research in Biomedicine and Health, University of Split School of Medicine, Šoltanska 2A, Split, 21000, Croatia, 385 21 557 820; 2 Department of Prosthodontics, University of Split School of Medicine, Split, Croatia; 3 Computer Science Department, Universitat de València, Valencia, Spain; 4 Department of Didactics of Mathematics, Universitat de València, Valencia, Spain

**Keywords:** usability, quality of evidence, GRADE, cognitive load, decision-making, Grading of Recommendations Assessment, Development, and Evaluation

## Abstract

**Background:**

Summary of findings (SoF) tables are widely used in systematic reviews and clinical practice guidelines to present evidence about health care interventions in a concise and transparent format. Although developed to improve accessibility and interpretation of evidence, previous studies have shown that users often experience difficulties understanding statistical information, certainty ratings, and the relationships between outcomes and treatment effects. Limited research has explored how different groups of users cognitively interact with SoF tables while solving evidence interpretation tasks, particularly when table complexity increases.

**Objective:**

This study aimed to investigate how different types of users—Grading of Recommendations Assessment, Development, and Evaluation (GRADE) or Cochrane experts, practicing clinicians, and health care users—interact with SoF tables of varying complexity while answering intervention-related questions, and to examine differences in search behavior, navigation patterns, and task performance.

**Methods:**

We used the Read&Learn tool (ERI-Lectura and LAIA-UV) in an online, single-session study to evaluate participants’ interactions with SoF tables. Participants (n=120; 40 per group) accessed preselected SoF tables via a secure link and unique login. Participants completed tasks involving 4 SoF tables with increasing complexity. Specific table cells were blurred and had to be clicked to reveal information. Outcomes included the number of correct answers, total time spent on tasks, number of table cells visited, number of target and nontarget cells, and question-reading behavior.

**Results:**

Simpler SoF tables with a small number of outcomes and single target cells were correctly interpreted by most participants, regardless of their expertise. As table complexity and task demand increased, all participant groups demonstrated reduced performance and less efficient navigation patterns. Experts generally performed better than clinicians and health care users, particularly by spending less time reading nontarget cells and visiting fewer irrelevant table elements. Nevertheless, even experts experienced difficulties with tasks requiring synthesis and interpretation across multiple table cells. Questions requiring comparison and integration of information across outcomes resulted in the highest rates of incorrect responses in all groups. Heatmaps of the number of clicks and time spent on tables demonstrated that experts used more targeted search strategies, whereas clinicians and health care users explored a larger number of nontarget cells and spent more time navigating the tables. The most complex SoF tables produced the highest cognitive demands for all groups, suggesting that increasing element interactivity and information density substantially affect usability.

**Conclusions:**

SoF tables remain cognitively demanding even for experienced users of evidence synthesis. Increasing table complexity appears to reduce users’ ability to identify, interpret, and synthesize relevant information. These findings suggest that current SoF formats may impose substantial intrinsic and extraneous cognitive load, particularly for nonexpert audiences. Future development of SoF tables should prioritize clearer presentation of clearer outcomes, inclusion of absolute alongside relative effects, and interactive or user-centered formats that support evidence navigation and interpretation.

## Introduction

Systematic reviews represent the fundamental unit of knowledge translation, providing a rigorous synthesis of evidence to support informed health care decisions [1]. However, the exponential growth of medical literature has led to significant information overload, with thousands of new reviews published annually [2]. In clinical settings, health care professionals operate under severe time constraints, often spending less than 2 minutes pursuing an answer to a clinical question [3]. Consequently, many stakeholders struggle to engage with lengthy, data-dense technical reports that lack necessary contextual details [1].

Comprehension of research evidence is further hindered by the fact that text-based information regarding treatment effects is often interpreted inconsistently by different users [4]. Furthermore, even highly trained health professionals and medical students frequently exhibit low health numeracy skills, struggling with the interpretation of essential statistical concepts such as risk ratios, CIs, and probabilistic reasoning [5]. Recent linguistic analyses also indicate that scientific literature is becoming increasingly difficult to read over time due to the proliferation of technical jargon and heightened cognitive complexity [6].

To bridge the gap between scientific research and clinical practice, the Grading of Recommendations Assessment, Development, and Evaluation (GRADE) Working Group promotes the use of summary of findings (SoF) tables [7]. These tables provide a structured, transparent, and concise summary of key results, presenting the magnitude of relative and absolute effects for patient-important outcomes alongside a rating of the certainty of the evidence [8]. It has been demonstrated that including an SoF table in a review significantly improves a reader’s ability to find critical information faster, reducing search time from an average of 4 minutes to just 90 seconds, and doubling comprehension accuracy compared with narrative text alone [9].

In the past several years, research has increasingly focused on optimizing SoF table formats to better serve diverse stakeholders. Recent randomized trials have shown that alternative GRADE SoF formats incorporating number-needed-to-treat and absolute risk differences significantly improve risk quantification compared with the traditional format [8]. Similarly, co-design workshops have highlighted a “less is more” preference among guideline developers, emphasizing the need for standardized narrative descriptions of results to facilitate “gist processing,” where individuals encode and retrieve essential, bottom-line meaning of information rather than its precise numerical surface details [1]. Despite these optimizations, evidence synthesis summaries, particularly in specialized fields like oncology, still face challenges with low readability and often provide unclear conclusions for lay audiences [6].

A critical unresolved issue is how different categories of readers actually navigate the complex visual field of an SoF table to interpret the presented data. In our previous study [9], we used the Read&Learn tool (ERI-Lectura and LAIA-UV), which uses a “blurred interface” approach to quantify cognitive interaction by revealing content only when a specific cell is clicked. Our findings indicated that while medical students were predominantly answer-oriented, spending a median of 72% (IQR 259.9-588.3 s) of their time reading target cells, they frequently struggled with tasks requiring higher-level critical thinking, such as making practice recommendations based on complex outcomes.

Building on this work, in this study we investigated how different users cognitively interact with SoF tables: (1) GRADE and/or Cochrane experts, who are familiar with SoF tables; (2) practicing clinicians, who are expected to be familiar with the SoF tables in everyday work, mostly through clinical practice guidelines; and (3) health care users, who may encounter SoF tables when reading systematic reviews or clinical practice guidelines.

## Methods

### Setting

The study was conducted using the Read&Learn online platform [10] from February to June 2025. The study was preregistered in the Open Science Framework (OSF) [11].

### Participants

Three groups of participants were recruited by convenience sampling:

1. GRADE group members and/or producers of Cochrane systematic reviews, contacted through their correspondence information in relevant publications or through our contacts within Cochrane or GRADE.
2. Practicing physicians: doctors from the University Hospital of Split, Split, Croatia, having an MD or DMD degree and being proficient in English.
3. Health care users: any person who uses the health care system, recruited using the contacts of partner patient associations of Cochrane Croatia and personal contacts.

### Interventions

We used the online Read&Learn tool [10] to evaluate how participants interacted with the SoF tables while answering questions about the evidence on a health intervention. The participants received the link for the survey via email and a unique single-use username and password.

The intervention involved blurring specific table cells within a digital platform, requiring participants to click to reveal information. There were no time restrictions.

The participants were presented with 2 separate screens, one containing a question and a box for their answer, and the other displaying a SoF table with blurred cells (Multimedia Appendix 1). The first SoF table column with outcomes and the headings of other columns remained visible at all times. Clicking on a cell in the SoF table revealed its content, and an opened cell became blurred when another was clicked. Moreover, 2 authors (NV and NB) independently decided on the target cells for each question, without disagreements. After answering the questions, the system presented the next question and the same SoF table, with blurred cells.

We presented 4 different SoF tables (Multimedia Appendix 1):

First, SoF1 [12] served as a practice task to introduce participants to the structure and interaction format. It contained 2 outcomes and 2 corresponding questions (Q1 and Q2), designed to be simple and intuitive. Each question had its own single target table cell.

Second, SoF2 [12] included 3 outcomes and 3 open-ended questions (Q1-Q3), each with a clearly defined target cell. All answers could be directly retrieved from the table; no additional steps were required.

Third, SoF3 [13] represented a moderate level of complexity, with 7 outcomes and 7 questions (Q1-Q7): 2 open-ended questions and 3 multiple-choice questions followed by 2 reflective questions (“Now, review the comment provided in the table and compare it to your answer to evaluate whether your perspective aligns with the comment. Would you reconsider your response?,” “If yes, explain why.”). Moreover, 3 questions had 2 target cells each, while 2 questions relied on a single target cell. This table also required the interpretation of information across 7 target cells for reflective questions.

Fourth, SoF4 [14] was the most complex, including 4 outcomes and 8 questions (Q1-Q8; 2 open-ended questions, 4 multiple-choice questions, and 2 reflective comment questions, the same as for SoF3). There were 10 target cells in total: 2 questions relied on a single cell, and 4 questions required participants to identify and interpret information from 2 different cells. Questions in this table required a higher level of cognitive effort, as participants had to actively search, combine, and interpret data from multiple table cells to be able to give a correct response.

Face validity of the experimental set-up was tested by 5 researchers from the Center of Evidence-based Medicine. Minor changes to the wording of the questions were made.

### Outcomes

The primary outcome measure was the number of correct answers to the knowledge questions about the information in each SoF table (Multimedia Appendix 1).

Secondary outcome measures related to their search behavior: (1) total number of table cells visited during search, (2) number of target or nontarget table cells visited, (3) number of times reading the question statement, and (4) number of times searching in text for an answer. Time variables (in seconds) were (1) total time spent on the table, from beginning to the end; (2) total time for initial reading; (3) time spent on quiz questions; (4) total time reading cells during search; (5) total time reading target or nontarget segments during search; and (6) total time reading questions.

Heatmaps were created for the average times (in seconds) spent reading SoF table cells and the number of clicks (readings) for each SoF table cell.

The participants also completed group-specific demographic questionnaires tailored to their background. All participants self-reported their gender. For the purposes of analysis, responses were categorized as female or male.

### Sample Size

We hypothesized that the expected difference between the groups would be 4 points on a scale from 0 to 16 for the number of correctly answered questions, with an SD of 5. Based on those parameters, with 80% power and a 5% α error rate, we used an online sample size calculator (Select Statistical Services sample size calculator) to calculate the sample size at 25 participants per group.

### Blinding

Participants, those administering the interventions, and those assessing the outcomes were aware of the study condition assignments, reflecting the quasi-experimental design. Data analysis was performed in a blinded fashion.

### Statistical Analysis

Each participant was a unit of analysis. Only the complete responses were included in the analysis. We assessed data normality using the Shapiro-Wilk test and presented the data as medians with IQR. We used the Kruskal-Wallis test and the Conover post hoc test to test the differences between the 3 groups. The significance level was set at *P*<.05. We performed the statistical analyses using MedCalc (v 23.2.1; MedCalc Software Ltd).

The results of the study are reported according to the Transparent Reporting of Evaluations with Nonrandomized Designs (TREND) reporting guidelines [15].

### Ethical Considerations

The study was performed in accordance with relevant regulations and guidance on social science research involving human participants. The Ethics Committee of the University of Split School of Medicine approved the study on October 24, 2024 (Document Class: 029-01/24-02/0001, registration 2181-198-03-04-24-0101). Only the lead researcher (NV) had access to participants’ personal information (name and email) during the conduct of the study for the purpose of correspondence. All others, including other researchers, only received data once it had been anonymized. The researcher retrieving the data from the Read&Learn platform had no access to participants’ identities. The output from the platform was fully anonymous, and the platform does not link the results with the entry codes.

Participants did not receive financial compensation or other incentives for participation in the study.

## Results

**Figure 1.**
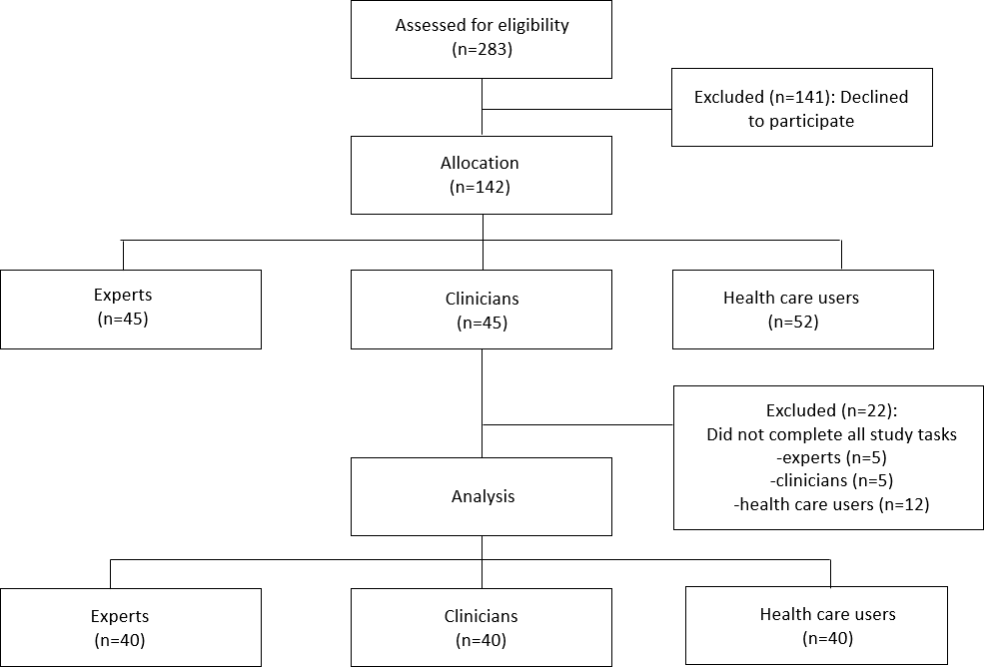
Participant flow diagram.

A total of 283 invitations were sent via email to identified contacts (88 GRADE and/or Cochrane experts, 48 clinicians, and 147 health care consumers; Figure 1). About half of the invited participants took part in the study (142/283, 50.2%): 45/48 (94%) clinicians, 45/88 (51%) experts, and 52/147 (35%) health care users. After the experiment, 22 participants were excluded from the analysis for incomplete answers (5 experts, 5 clinicians, and 12 health care users). Data from 40 experts, 40 clinicians, and 40 health care users were analyzed.

All groups (Table 1) had more female participants. Among experts, the median age was around 40 (IQR 33.0-47.2) years and the median research experience was 12 (IQR 7.0-17.0) years. Most (30/40, 75%) were affiliated with Cochrane work; almost all reported previous experience working on systematic reviews. For clinicians, the median age was 30 (IQR 28.0-32.5) years, with a median of 5 (IQR 3.0-7.0) years of professional experience. More than half were residents (34/40, 85%), and came from different medical specialties (Multimedia Appendix 1). More than half of them reported using guidelines in their clinical practice, and most had no previous experience in creating systematic reviews or developing clinical practice guidelines. The median age of health care users was 39.5 (IQR 30.7-45.5) years, with varied educational backgrounds, mostly at the graduate and postgraduate levels. About a third reported some formal education in biomedicine and health.

**Table 1.** Demographic characteristics of study participants.

Characteristics	Statistical value
Experts (n=40)	
Age (y), median (IQR)	42.5 (33.0-47.2)
Self-reported sex, n (%)	
Female	24 (60%)
Male	16 (40%)
Last degree, n (%)	
PhD	26 (65%)
MD	12 (30%)
Master’s degree	1 (2.5%)
Did not disclose	1 (2.5%)
Years of research experience (median, 95% CI)	12.0 (7.0-17.0)
Group affiliation, n (%)	
Cochrane group	30 (75%)
GRADE^a^ working group	10 (25%)
Worked on systematic reviews for health interventions or clinical practice guidelines, n (%)	
Yes	38 (95%)
No	2 (5%)
Clinicians (n=40)	
Age (y), median (95% CI)	30 (28.0-32.5)
Self-reported sex, n (%)	
Female	24 (60%)
Male	16 (40%)
Current professional position, n (%)	
Resident	34 (85%)
Specialist	4 (10%)
Subspecialist	1 (2.5%)
Did not disclose	1 (2.5%)
Years of professional experience (y), median (95% CI)	5.0 (3.0-7.0)
Degree in science (MSc, PhD), n (%)	
Yes	2 (5%)
No	38 (95%)
Actively using clinical guidelines in practice, n (%)	
Yes	27 (67%)
No	13 (33%)
Participated in a systematic review of health interventions or the creation of clinical practice guidelines, n (%)	
Yes	3 (8%)
No	37 (92%)
Health care users (n=40)	
Age (y), median (95% CI)	39.5 (30.7-45.5)
Self-reported sex, n (%)	
Female	28 (70%)
Male	12 (30%)
Level of education, n (%)	
Graduate	17 (42.5%)
Postgraduate	11 (27.5%)
Undergraduate	10 (25%)
Secondary school	2 (5%)
Formal education in the field of biomedicine and health, n (%)	
Yes	14 (34.1%)
University studies	11
Patient association	1
High school	2
No	27 (67%)
Professional health training?, n (%)	
Yes	6 (15%)
No	34 (85%)
Used health care services in the last ten years (for health check-ups or for treatment)?, n (%)	
Yes	30 (90%)
No	10 (10%)

a GRADE: Grading of Recommendations Assessment, Development, and Evaluation.

SoF1 (Table 2) had 12 cells in total and 2 knowledge questions, each with a single target cell. The median time spent by all groups on answering the questions was under 2 minutes (IQR 21.1-144.2 s), and the questions were correctly answered by most participants (115/120, 95%). There were no significant differences between the groups across all 13 interaction parameters. The heatmap of interactions (Multimedia Appendix 1) showed that all 3 groups were mostly answer-oriented for both questions. Out of 12 table cells in total, every cell was opened over 20 times. Experts read all table cells, whereas clinicians and health care consumers had unread cells (4 and 2 cells, respectively).

**Table 2.** Summary statistics for interaction parameters for the first summary of findings table by experts, clinicians, and health care users^a^.

Parameter	Experts (n=40), median (IQR)	Clinicians (n=40), median (IQR)	Health care users (n=40), median (IQR)	*P* value^b^
Number of correct answers (maximum 2 points)	2.0 (2.0‐2.0)	2.0 (2.0‐2.0)	2.0 (2.0‐2.0)	.89
Total time (s) spent on the table, from the beginning to the end	41.9 (11.1-107.1)	110.4 (26.8-177.6)	53.8 (21.1-154.9)	.18
Time (s) spent on initial reading^c^	0.0 (0.0-7.4)	0.0 (0.0-19.5)	0.5135 (0.0-23.9)	.46
Time (s) spent on quiz questions	52.4 (23.7-111.1)	68.9 (31.1-107.6)	52.6 (34.4-117.4)	.77
Total number of table cells visited	3.0 (2.0-12.0)	4.5 (2.0-15.5)	7.0 (2.0-15.0)	.38
Total time (s) reading table cells during search	7.3 (2.3-14.7)	7.8 (2.9-19.1)	8.3 (4.5-20.9)	.53
Total time (s) spent on reading target cells	8.0 (2.3-18.5)	8.7 (4.2-19.5)	8.8 (5.4-20.9)	.49
Number of target cells visited	3.0 (2.0-4.0)	3.0 (2.0-5.0)	3.5 (2.0-6.0)	.33
Total time (s) spent on reading nontarget cells	0.5 (0.0-8.2)	2.4 (0.0-21.1)	5.2 (0.0-28.3)	.28
Number of nontarget cells visited	0.5 (0.0-8.0)	1.0 (0.0-11.3)	4.0 (0.0-10.7)	.31
Time (s) reading the question statement	13.1 (3.6-25.4)	19.6 (10.8-32.1)	13.9 (6.9-20.2)	.25
Number of times reading the question statement	3.5 (2.0-5.0)	3.5 (2.0-4.0)	3.0 (2.0-4.0)	.40
Number of times searching in text for an answer	2.0 (2.0-4.0)	2.0 (2.0-3.2)	2.0 (2.0-3.0)	.91

a The first summary of findings table in the test [12] consisted of 12 interactive cells, each of which required the user to click to reveal the information. There were 2 questions, each was associated with one target cell in the table. The table displayed 6 column headings and 2 outcomes, all of which remained visible throughout the task. Participants had continuous access to both the descriptive text above the table and the footnotes below it.

b Kruskal-Wallis test.

c Time spent on the initial reading of the summary of findings table, after the start of the task but before switching to the question panel.

SoF2 (Table 3) had 18 cells in total and 3 knowledge questions. Experts spent significantly less time reading nontarget cells and question statements and visited the fewest total number of table cells and nontarget cells. Questions 1‐3 had only 1 target segment each for the 3 questions, and most of the participants correctly answered them (112/120, 93% for Q1; 107/120, 89% for Q2; and 111/120, 92% for Q3). The heatmap (Multimedia Appendix 1) showed that the participants were focused on answering specific questions. The experts spent the shortest average time on nontarget cells, and the average reading time increased with less experience of the participant group with the SoF tables.

**Table 3.** Summary statistics for interaction parameters for the second summary of findings table by experts, clinicians, and health care users^a^.

Parameter	Experts (n=40), median (IQR)	Clinicians (n=40), median (IQR)	Health care users (n=40), median (IQR)	*P* value^b^
Number of correct answers (maximum 3 points)	3.0 (3.0‐3.0)	3.0 (3.0‐3.0)	3.0 (3.0‐3.0)	.09
Total time (s) spent on the table, from the beginning to the end	47.4 (16.8-79.0)	72.9 (37.5-112.7)	64.8 (25.9-99.1)	.08
Time (s) spent on initial reading^c^	0.0 (0.0‐0.0)	0.0 (0.0‐0.7)	0.0 (0.0-2.7)	.43
Time (s) spent on quiz questions	101.4 (45.7-136.5)	124.5 (63.0-171.3)	99.1 (63.0-176.8)	.44
Total number of table cells visited	4.0 (3.0-9.0)^d^	7.0 (3.7-12.5)	6.5 (4.0-21.0)	.03
Total time (s) reading table cells during search	13.2 (4.3-30.5)	18.6 (7.1-35.8)	17.8 (8.5-38.2)	.59
Total time (s) spent on reading target cells	14.6 (3.4-28.4)	16.7 (7.1-32.7)	14.8 (7.4-34.7)	.34
Number of target cells visited	3.0 (3.0-5.2)	4.0 (3.0-6.0)	4.0 (3.0-7.2)	.22
Total time (s) spent on reading nontarget cells	0.0 (0.0-5.4)^d^	5.4 (0.0-17.4)	5.2 (0.0-28.3)	.01
Number of nontarget cells visited	0.0 (0.0-2.2)^d^	2.5 (0.0-7.2)	4.0 (0.0-10.8)	.01
Time (s) reading the question statement	20.4 (10.7-31.0)^d^	32.5 (19.3-55.1)	37.1 (16.3-48.2)	.03
Number of times reading the question statement	4.0 (3.0-6.0)	5.0 (3.0-6.0)	4.0 (3.0-6.0)	.71
Number of times searching in text for an answer	4.0 (3.0-5.0)	4.0 (3.0-5.2)	4.0 (3.0-5.2)	.90

a The second summary of findings table in the test [12] consisted of 18 interactive cells, each of which required the user to click to reveal the information. There were 3 questions, each was associated with 1 target cell in the table. The table displayed 6 column headings and 3 outcomes, all of which remained visible throughout the task. Participants had continuous access to both the descriptive text above the table and the footnotes below it.

b Kruskal-Wallis test.

c Time spent on initial reading of the summary of findings table after the start of the task before switching to the question.

d Significantly different versus other groups; Conover post hoc test, *P*=.04 vs clinicians and *P*=.01 vs health care users.

SoF3 (Table 4) had 42 cells in total and 5 knowledge questions. Overall, most of the participants correctly answered the questions (97/120, 81%), with Q3 and Q6 consistently having the most incorrect answers (22/120, 18% for Q3 and 22/120, 19% for Q6). Experts had significantly more correct answers than clinicians and health care users. They also outperformed the other 2 groups by spending the least amount of time solving the questions, reading nontarget cells, and reading the question statement, as well as having the lowest number of visits to total cells or nontarget cells. In reflexive questions, 4 (3.3%) participants indicated they would change their previous answer to Q5 after reading the comment, and 9 (7.5%) participants did so for Q7. For the 5 knowledge questions, the heatmap (Multimedia Appendix 1) showed that all 3 groups spent the most time and had the highest number of readings on target cells containing the correct answer. Across all 3 groups, the nontarget cells that were in the same row as the target cell were next in terms of the number of times the participants opened them and the average time they were reading them. The heatmap also showed that all 3 groups were opening and reading the “Comments” column throughout the solving of the table, even though they were only asked to open it during the 2 reflexive questions.

**Table 4.** Summary statistics for interaction parameters for the third summary of findings table by experts, clinicians, and health care users^a^.

Parameter	Experts (n=40), median (IQR)	Clinicians (n=40), median (IQR)	Health care users (n=40), median (IQR)	*P* value^b^
Number of correct answers (maximum 5 points)	5.0 (5.0‐5.0)^c^	5.0 (4.0‐5.0)	4.0 (3.7-5.0)	.01
Total time (s) spent on the table, from the beginning to the end	107.2 (52.7-189.4)^d^	222.2 (73.4-329.6)	178.4 86.5-318.1)	.04
Time (s) spent on initial reading^e^	0.0 (0.0‐0.0)	0.0 (0.0‐0.0)	0.0 (0.0‐5.8)	.08
Time (s) spent on quiz questions	249.8 (126.1-428.2)	441.6 (193.6-630.8)	369.1 (186.5-526.1)	.12
Total number of table cells visited	30.5 (24.0-38.2)^d^	37.0 (26.0-52.2)	38.0 (29.0-48.5)	.03
Total time (s) reading table cells during search	57.7 (31.5-102.7)	86.1 (30.8-154.7)	72.5 (37.9-119.9)	.66
Total time (s) spent on reading target cells	43.8 (16.5-66.5)	40.8 (18.1-79.2)	42.5 (23.8-63.9)	.82
Number of target cells visited	16.0 (14.0-20.2)	18.0 (14.0-25.5)	17.0 (13.7-21.2)	.36
Total time (s) spent on reading non-target cells	19.4 (12.7-43.9)^c^	43.1 (15.8-83.6)	45.4 (24.3-69.2)	.02
Number of nontarget cells visited	14.0 (10.0-18.2)^e^	19.0 (12.7-29.0)	21.5 (14.7-30.2)	.003
Time (s) reading the question statement	59.6 (25.1-84.1)^d^	99.6 (57.7-136.6)	107.2 (39.1-162.7)	.01
Number of times reading the question statement	12.0 (8.0-15.0)	15.0 (9.7-17.2)	13.0 (9.0-15.0)	.20
Number of times searching in text for an answer	8.0 (7.0-10.0)	8.0 (7.0-12.0)	8.0 (7.0-11.0)	.60

a The third summary of findings table in the test [13] consisted of 42 interactive cells, each of which required the user to click to reveal the information. There were 7 questions, 4 had only 1 target cell, the rest of the questions had 2 target cells. The table displayed 6 column headings and 7 outcomes, all of which remained visible throughout the task. Participants had continuous access to both the descriptive text above the table and the footnotes below it.

b Kruskal-Wallis test.

c Significantly different vs health care users; Conover post hoc test, *P*=.004.

d Significantly different versus other groups; Conover post hoc test, *P*=.04 vs clinicians, *P*=.02 vs health care users.

e Time spent on initial reading of the summary of findings table after the start of the task before switching to the question.

**Figure 2.**
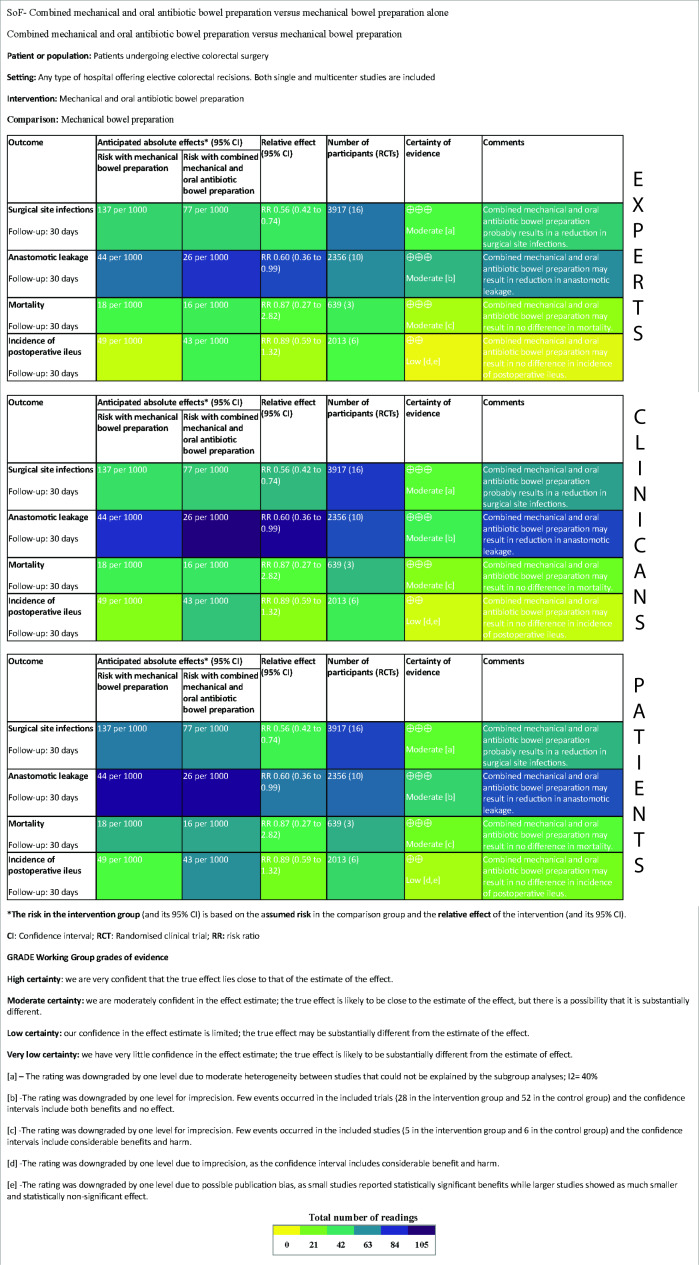
Heatmap of the total number of table cell readings (clicks) for the fourth summary of findings table.

**Figure 3.**
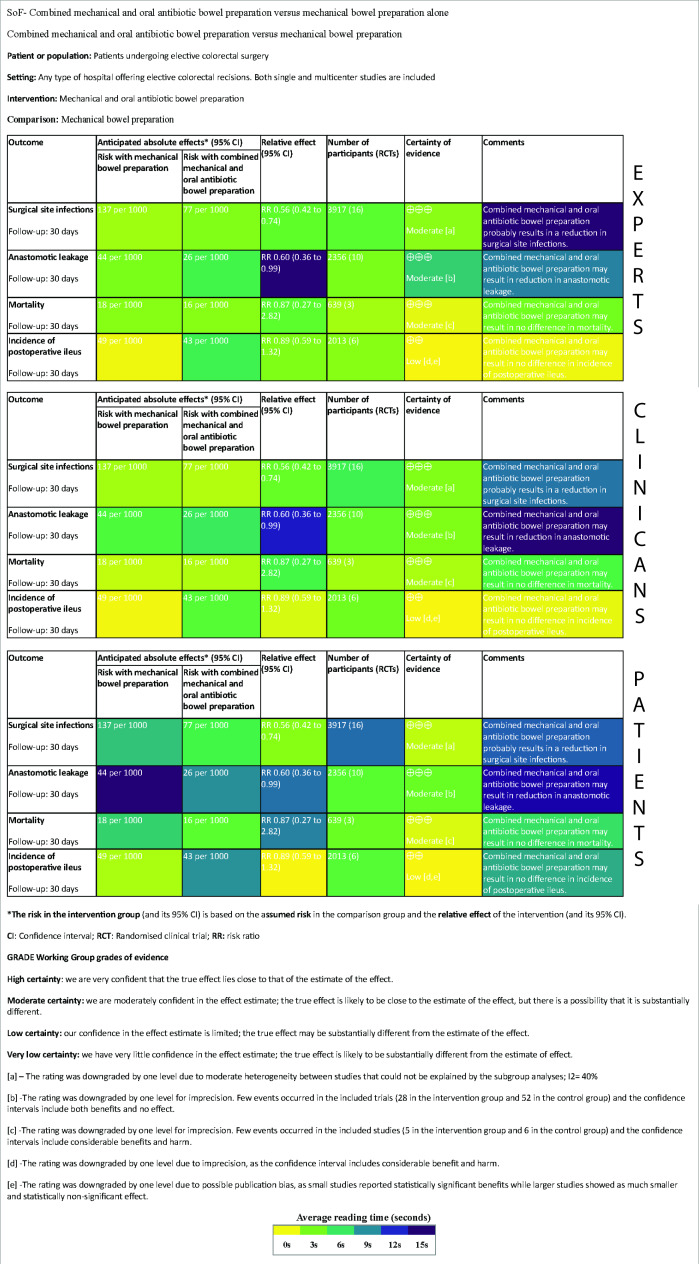
Heatmap of the average reading time for the fourth summary of findings table.

SoF4 (Table 5) had 24 table cells in total and 6 knowledge questions. Overall, all groups performed worse for questions with more than 1 target cell, compared with the other 3 tables. The question with the second largest number of wrong answers was Q3 (31/120, 26% of participants), 10% (12/120) gave a wrong answer to Q4, and 23% (28/120) to Q5. Q7 had the highest number of wrong answers across all four tables (41/120, 34% of total participants). For 2 reflexive questions, 9 (7.5%) participants for Q6 and 7 (5.8%) participants for Q8 said they would change their answer. For each question in this table, health care users had the lowest number of correct answers and spent significantly more time on the initial reading than the other 2 groups. Experts spent less time reading the question statement than the other groups and spent significantly less total time on the table, from beginning to the end, compared with clinicians, and visited fewer nontarget cells than health care consumers. The heatmap of interactions confirmed that this was the most challenging table for all participants, both in terms of the number of readings (clicks; Figure 2) and average time of reading table cells (Figure 3). Not all cells with the highest number of visits and average reading time were target cells. Health care users spent more time reading the Comment section than the other 2 groups for unrelated questions.

**Table 5.** Summary statistics for interaction parameters for the fourth summary of findings table by experts, clinicians, and health care users^a^.

Parameter	Experts (n=40), median (IQR)	Clinicians (n=40), median (IQR)	Health care users (n=40), median (IQR)	*P* value^b^
Number of correct answers (maximum 6 points)	5.0 (4.0-6.0)	6.0 (4.7-6.0)^c^	5.0 (44.0-6.0)	.05
Total time (s) spent on the table, from the beginning to the end	96.2 (57.9-157.9)	127.3 (88.4-187.7)	110.7 (68.0-163.4)	.18
Time (s) spent on initial reading^d^	0.0 (0.0‐0.0)	0.0 (0.0‐0.0)	0.0 (0.0-6.4)^c^	.005
Time (s) spent on quiz questions	225.1 (144.4-322.0)^e^	337.5 (245.3-468.4)	281.7 (153.9-407.8)	.04
Total number of table cells visited	29.0 (25.7-34.5)	34.5 (24.7-44.2)	32.5 (24.0-45.0)	.24
Total time (s) reading table cells during search	53.1 (33.2-81.7)	64.9 (43.1-103.5)	46.4 (37.6-92.2)	.51
Total time (s) spent on reading target cells	33.1 (22.4-64.6)	42.1 (27.1-70.8)	37.8 (27.8-58.3)	.70
Number of target cells visited	20.5 (16.0-24.0)	23.5 (15.7-30.0)	21.0 (15.0-27.2)	.47
Total time (s) spent on reading nontarget cells	13.4 (9.8-19.9)	22.1 (13.9-28.4)	17.6 (13.3-38.3)	.70
Number of nontarget cells visited	9.0 (7.0-12.0)^f^	10.5 (8.7-15.5)	13.5 (10.0-19.0)	.003
Time (s) reading the question statement	56.9 (30.1-80.5)^c^	84.6 (52.6-128.2)	88.6 (41.6-132.5)	.008
Number of times reading the question statement	12.0 (9.7-15.0)	14.0 (10.7-16.2)	13.0 (10.0-17.2)	.35
Number of times searching in text for an answer	9.0 (8.0-10.0)	9.0 (8.0-12.0)	9.0 (7.0-11.0)	.55

a The fourth summary of findings table in the test [14] consisted of 24 interactive cells, each of which required the user to click to reveal the information. There were 8 questions, 4 questions had only 1 target cell, and 4 questions had 2 target cells. The table displayed 6 column headings and 4 outcomes, all of which remained visible throughout the task. Participants had continuous access to both the descriptive text above the table and the footnotes below it.

b Kruskal-Wallis test.

c Significantly different versus other groups; Conover post hoc test, *P*=.04 vs clinicians, *P*=.02 vs health care users.

d Time spent on initial reading of the summary of findings table after the start of the task before switching to the question.

e Significantly different vs clinicians; Conover post hoc test, *P*=.01.

f Significantly different vs health care users; Conover post hoc test, *P*=.001.

## Discussion

### Principal Findings

This study investigated how experts in evidence synthesis, clinicians, and health care users cognitively interacted with SoF tables of increasing complexity while answering intervention-related questions. Overall, we found that simpler SoF tables with clearly identifiable target information were interpreted successfully by most participants regardless of expertise. However, as the complexity of the tables and the cognitive demand of the tasks increased, performance declined across all groups. Experts demonstrated more targeted search behavior and more efficient navigation patterns, but even they experienced difficulties with tasks requiring interpretation and synthesis across multiple table elements. These findings align with our study objective of examining how different user groups search for, process, and interpret information presented in SoF tables.

We presented SoF tables with increasing complexity and increasing difficulty of knowledge questions, which increased the cognitive load presented to the study participants. Cognitive load, defined as the mental effort required to process information within working memory [16], is relevant in the context of our study, as interpreting SoF tables involves complex information that can be difficult to process, especially for health care users. Cognitive load is influenced by the complexity of the task (intrinsic cognitive load), the way information is presented (extraneous cognitive load), and the effort involved in building new knowledge structures (germane cognitive load) [16].

Intrinsic load is the inherent level of difficulty associated with learning the material or task itself [17]. All SoF tables contained statistical data, effect sizes, CIs, GRADE certainty ratings, and outcome comparisons, whose understanding required some level of statistical and methodological knowledge. The intrinsic load of SoF3 and SoF4 was high enough to challenge all groups, which likely leveled the performance across experts and nonexperts. Consequently, experts showed advantages in only 4-5 out of the 13 parameters, suggesting that previous knowledge could mitigate but not fully overcome the cognitive demands imposed by tasks with high element interactivity.

Extraneous load mainly comes from how the information is presented and the instructional design, which can raise cognitive load without improving learning [17]. Poorly designed tables (eg, unclear headings, jargon, dense text, or inconsistent formatting) increase extraneous load [17]. The varying number of cells, outcomes, and proportion of target to nontarget information in our study likely added to extraneous load, as participants had to search through irrelevant content. In addition, the Read&Learn platform required participants to reveal 1 table cell at a time, while previously opened cells became blurred again. This likely increased working memory demands because participants had to retain previously viewed information while comparing outcomes and synthesizing answers across multiple table cells. Such conditions differ from real-world reading of SoF tables, where multiple data points remain simultaneously visible and can be rapidly compared through eye movement. This may explain why performance differences between groups were limited, since higher extraneous demands made the tables more difficult to navigate, regardless of the expertise.

Our findings also suggest that comprehension of SoF tables should not be viewed as a single construct, but rather as a combination of multiple cognitive processes, including information retrieval, interpretation, comparison, recall, and synthesis. Participants generally performed well on simpler tasks requiring identification of a single target value, but had substantially more difficulty with questions requiring comparison of multiple outcomes or integration of statistical and textual information. This finding is consistent with previous literature [4,18] showing that different evidence presentation formats may support some cognitive tasks better than others.

Recent research in digital information processing and cognitive load has similarly shown that increasing informational complexity and element interactivity can negatively affect comprehension and decision-making performance, particularly in cognitively demanding environments [19].

Germane load refers to the mental effort learners invest in processing and working with the material in ways that support understanding and promote learning [20]. Giving supportive information before practice can significantly reduce unnecessary searching that does not contribute to learning [21]. In our study, the first SoF table served the purpose of familiarizing participants with the task format rather than testing performance differences. By allowing participants to focus on understanding how the tables functioned and how to navigate them, this table primarily served to prepare them for the subsequent, more complex tasks. The third and fourth tables included more complex questions, requiring participants to draw their own conclusions rather than merely locate information. This design element allowed us to observe how increased cognitive demand influenced problem-solving and interpretation across different user groups. Taken together, our findings suggest that many participants, including experts, struggle when required to move beyond basic understanding and application, especially when confronted with tasks outside their narrow domain of expertise. Experts nevertheless demonstrated more focused navigation patterns, including fewer visits to nontarget cells and shorter time spent searching irrelevant information. However, these differences likely reflect not only familiarity with SoF tables but also broader evidence-literacy skills acquired through methodological training and experience in evidence synthesis. Therefore, the observed differences should not be interpreted solely as evidence of superior usability of the SoF format itself, but rather as reflecting an interaction between table design and user expertise. These findings suggest that nonexpert users may require additional guidance, training, or alternative evidence presentation formats to efficiently identify and interpret the most relevant information within complex evidence summaries.

Although this study focuses on SoF tables, similar challenges have been observed in other forms of health communication. For example, Sander et al [22] found that design and presentation features of hospital report cards influenced users’ comprehension, and van der Mee et al [23] reported that the way laboratory results are displayed affects patient understanding. These parallels suggest that improving the clarity and usability of evidence presentation is a broader concern in health care communication, with SoF tables representing one particularly high-stakes context where comprehension directly informs decisions.

### Limitations

Although all participants used the same digital tool (Read&Learn), none were familiar with it beforehand, which could have impacted their interaction with the task. The Read&Learn platform had a double-screen setup, where the participants had to switch between 2 screens. This required them to remember the question they read while searching for the answer in the table, increasing cognitive demands. Also, because participants solved the experiment online without supervision, we cannot tell if they focused entirely on the tasks while solving a question, or if they experienced outside distractions that affected their reaction.

Another important limitation is that the selective cell-uncovering methodology does not fully replicate real-world interaction with SoF tables. Requiring users to click on cells to reveal information may have restricted natural search behavior and increased task difficulty compared with fully visible tables. This approach likely emphasized memory and sequential processing more strongly than ordinary reading conditions, particularly for questions requiring comparison of multiple values across outcomes. Therefore, the findings should be interpreted as reflecting cognitive interaction patterns under controlled experimental conditions rather than direct simulation of real-world SoF table use.

Differences in participants’ experience with technology and data comprehension may have also influenced performance across groups. Additionally, the wording of questions may have influenced the overall participants’ search strategies and patterns of opening cells within the tables, potentially affecting how information was accessed and processed.

The study may also have been underpowered to detect smaller intergroup differences for some secondary behavioral outcomes, particularly given the variability in time-based interaction measures. Therefore, nonsignificant findings should be interpreted cautiously.

Because our expert groups included both international and Croatian professionals, the results for experts are likely applicable to a wider population. Other groups were mainly local, and findings for those participants may not fully apply to people in different regions or settings. Furthermore, the health care user sample was relatively highly educated and therefore may not represent the broader population of health care users. Participation in the study required computer use and interaction with an online platform, which limited recruitment to individuals with sufficient computer literacy and likely introduced selection bias. Additionally, all study materials and tasks were presented in English, requiring participants to have adequate English-language proficiency. Despite these characteristics, health care users still experienced substantial difficulties with more complex tasks, suggesting that interpretation challenges may be even greater in more representative general populations.

### Conclusions

Our findings suggest that the increasing complexity of SoF tables substantially affects users’ ability to efficiently identify, interpret, and synthesize evidence, even among experts familiar with evidence synthesis methods. While experts demonstrated more focused and efficient search strategies, expertise alone was insufficient to fully overcome the cognitive demands imposed by complex evidence summaries.

Despite being designed for clarity, SoF tables remain challenging even for experts who use them regularly, highlighting the cognitive demands of interpreting complex evidence. SoF tables should prioritize a small set of critical outcomes and present absolute alongside relative effects with certainty ratings. More interactive formats may help users find information more easily and navigate complex tables efficiently. Future research should explore user-centered and adaptive evidence presentation formats that reduce unnecessary cognitive burden while preserving transparency and methodological rigor. This is particularly important for patients and the general public, allowing nonexperts to understand the implications of different interventions, compare options, and make informed decisions based on reliable evidence.

## Supplementary material

10.2196/86045 Multimedia Appendix 1 Supplementary material.

10.2196/86045 Checklist 1 TREND reporting checklist.

## Abbreviations

GRADE: Grading of Recommendations Assessment, Development, and Evaluation
OSF: Open Science Framework
SoF: summary of findings
TREND: Transparent Reporting of Evaluations with Nonrandomized Designs
