# Supplementary material for: Comparative Analysis of Expert, Clinician, and Health Care User Interactions With Summary of Findings Tables: Usability Study
Source: J Med Internet Res. 2026 Jul 15;28:e86045. doi: 10.2196/86045 (PMC13372215; doi:10.2196/86045)
Supplement: Multimedia Appendix 1 [file jmir-v28-e86045-s001.pdf]

Supplementary material

Comparative analysis of expert, clinician, and healthcare user interactions with Summary of Findings tables: a quasi-experimental study

Nina Vitlov, Nensi Bralić, Tina Poklepović Peričić, Daniel Garcia-Costa, Emilia López-Iñesta, Elena Álvarez-García, Francisco Grimaldo, Ana Marušić

Table of Contents

Figure S1. Example of the Read&Learn platform interface

Text S1. Four Summary of Findings (SoF) tables and the accompanying questions for each

Figure S2. Heatmap of total number of readings (clicks) for the first Summary of Findings (SoF) table, for expert group. The colors correspond to the total number of times participants clicked on each cell, from 0 (yellow) to 105 (dark purple)

Figure S3. Heatmap of total number of readins (clicks) for the first Summary of Findings (SoF) table, for clinicinas group. The colors correspond to the total number of times participants clicked on each cell, from 0 (yellow) to 105 (dark purple)

Fugure S4. Heatmap of total number of readings (clicks) for the first Summary of Findings (SoF) table, for healthcare users group. The colors correspond to the total number of times participants clicked on each cell, from 0 (yellow) to 105 (dark purple)

Fugure S5. Heatmap of average reading time for the first Summary of Findings (SoF) table for expert group. The colors correspond to the average time spent on the individual table cell, from 0 seconds (yellow) to 15 seconds (dark purple)

Figure S6. Heatmap of average reading time for the first Summary of Findings (SoF) table for clinician group. The colors correspond to the average time spent on the individual table cell, from 0 seconds (yellow) to 15 seconds (dark purple)

Figure S7. Heatmap of average reading time for the first Summary of Findings (SoF) table for healthcare user group. The colors correspond to the average time spent on the individual table cell, from 0 seconds (yellow) to 15 seconds (dark purple)

Figure S8. Heatmap of total number of reading time (clicks) for the second Summary of Findings (SoF) table, for the expert group. The colors correspond to the total number of times participants clicked on each cell, from 0 (yellow) to 105 (dark purple)

Figure S9. Heatmap of total number of readings (clicks) for the second Summary of Findings (SoF) table, for the clinician group. The colors correspond to the total number of times participants clicked on each cell, from 0 (yellow) to 105 (dark purple)

Figure S10. Heatmap of total number of readings (clicks) for the second Summary of Findings (SoF) table, for the healthcare user group. The colors correspond to the total number of times participants clicked on each cell, from 0 (yellow) to 105 (dark purple)

Figure S11. Heatmap of average reading time for the second Summary of Findings (SoF) table for the expert group. The colors correspond to the average time spent on the individual table cell, from 0 seconds (yellow) to 15 seconds (dark purple)

Figure S12. Heatmap of average reading time for the second Summary of Findings (SoF) table for the clinician group. The colors correspond to the average time spent on the individual table cell, from 0 seconds (yellow) to 15 seconds (dark purple)

Figure S13. Heatmap of average reading time for the second Summary of Findings (SoF) table for the healthcare user group. The colors correspond to the average time spent on the individual table cell, from 0 seconds (yellow) to 15 seconds (dark purple)

Figure S14. Heatmap of total number of readings (clicks) for the third Summary of Findings (SoF) table, for the experts groups. The colors correspond to the total number of times participants clicked on each cell, from 0 (yellow) to 105 (dark purple)

Figure S15. Heatmap of total number of readings (clicks) for the third Summary of Findings (SoF) table, for the clinician groups. The colors correspond to the total number of times participants clicked on each cell, from 0 (yellow) to 105 (dark purple)

Figure S16. Heatmap of total number of readings (clicks) for the third Summary of Findings (SoF) table, for the healthcare user groups. The colors correspond to the total number of times participants clicked on each cell, from 0 (yellow) to 105 (dark purple)

Figure S17. Heatmap of average reading time for the third Summary of Findings (SoF) table for the expert groups. The colors correspond to the average time spent on the individual table cell, from 0 seconds (yellow) to 15 seconds (dark purple)

Figure S18. Heatmap of average reading time for the third Summary of Findings (SoF) table for the clinicnas group. The colors correspond to the average time spent on the individual table cell, from 0 seconds (yellow) to 15 seconds (dark purple)

Figure S19. Heatmap of average reading time for the third Summary of Findings (SoF) table for the healthcare user group. The colors correspond to the average time spent on the individual table cell, from 0 seconds (yellow) to 15 seconds (dark purple)

Table S1. Distribution of medical specialties among clinicians (n = 40)

Figure S1. Example of the Read&Learn platform interface

Questions

SoF table- Fibrin based haemostatic agents versus non-fibrin-based haemostatic agents

Fibrin-based haemostatic agents compared with non-fibrin-based haemostatic agents reducing intraoperative blood loss and improving outcomes in adult liver surgery

Population: adults undergoing liver resection for cancer or benign disease

Setting: clinical setting in liver resection unit

Intervention: fibrin-based haemostatic agents applied to resection surface

Comparison: non-fibrin-based haemostatic agents applied to resection surface

Outcome	Anticipated risk difference (95% CI)		Relative effect (95% CI)	Number of participants (RCTs)	Certainty of evidence	Comments
	Risk with non-fibrin-based haemostatic agents	Risk with fibrin-based haemostatic agents				
**Perioperative mortality**<br>Median follow-up 1.25 months (range 1 to 3 months)	[illegible]	[illegible]	[illegible]	[illegible]	[illegible]	[illegible]
**Serious adverse events**	[illegible]	[illegible]	[illegible]	[illegible]	[illegible]	[illegible]
**Reoperation**<br>Median follow-up 1.5 months (range 1 to 3 months)	[illegible]	[illegible]	[illegible]	[illegible]	[illegible]	[illegible]

***The risk in the intervention group** (and its 95% CI) is based on the **assumed risk** in the comparison group and the **relative effect** of the intervention (and its 95% CI).

CI: Confidence interval; **RCT**: Randomised clinical trial; **RR**: risk ratio

GRADE Working Group grades of evidence

High certainty: we are very confident that the true effect lies close to that of the estimate of the effect.

Moderate certainty: we are moderately confident in the effect estimate; the true effect is likely to be close to the estimate of the effect, but there is a possibility that it is substantially different.

Low certainty: our confidence in the effect estimate is limited; the true effect may be substantially different from the estimate of the effect.

Very low certainty: we have very little confidence in the effect estimate; the true effect is likely to be substantially different from the estimate of effect.

[a] – Downgraded one level for study limitations (overall high risk of bias).

[b] – Downgraded two levels for imprecision (few events and wide 95% CI including both benefit and harm).

[c] – Downgraded one level for imprecision (95% CI including both benefit and harm).

Text

Question box:

[illegible]

Text S1. Four Summary of Findings (SoF) tables and the accompanying questions for each

1) First SoF table

SoF table- Fibrin-based haemostatic agents versus no intervention or placebo

Fibrin-based haemostatic agents compared with no intervention or placebo for reducing intraoperative blood loss and improving outcomes in adult liver surgery

Population: adults undergoing liver resection for cancer or benign disease

Setting: liver resection unit

Intervention: fibrin-based haemostatic agents applied to resection surface

Comparison: haemostasis achieved through measures not including application of a topical agent (fibrin-based or otherwise)

**Outcome**	**Anticipated difference* (95% CI)**		**Relative effect (95% CI)**	**Number of participants (RCTs)**	**Certainty of evidence**	**Comments**
	**Risk with no intervention**	**Risk with fibrin-based haemostatic agents**				
**Perioperative mortality**<br>Median follow-up 1.5 months after liver resection (range 30 days to 6 months)	13 per 1000	34 per 1000 (12 to 89)	RR 2.58 (0.89 to 7.44)	782 (4)	⊕<br>Very low[a,b]	Defined as death, regardless of the cause, occurring within 30 days of surgery in or outside hospital
**Serious adverse events**<br>Median follow-up 1.5 months after liver resection (range 30 days to 6 months	435 per 1000	418 per 1000	RR 0.96 (0.88 to 1.05)	782 (4)	⊕<br>Very low[a,b]	Only 2/4 trials reported adverse events according to the definition provided in ICHGCP 2016.

***The risk in the intervention group** (and its 95% CI) is based on the a**ssumed risk** in the comparison group and the **relative effect** of the intervention (and its 95% CI).

CI: Confidence interval; **RCT**: Randomised clinical trial; **RR:** risk ratio

---

GRADE Working Group grades of evidence

High certainty: we are very confident that the true effect lies close to that of the estimate of the effect.

Moderate certainty: we are moderately confident in the effect estimate; the true effect is likely to be close to the estimate of the effect, but there is a possibility that it is substantially different.

Low certainty: our confidence in the effect estimate is limited; the true effect may be substantially different from the estimate of the effect.

Very low certainty: we have very little confidence in the effect estimate; the true effect is likely to be substantially different from the estimate of effect.

---

[a] – Downgraded one level for study limitations (overall high risk of bias).

[b] – Downgraded two levels due to serious imprecision (the optimal information size was not met (i.e. sample size < 1000), wide CI sin the result, few events, and the 95% CI includes both benefits and harms)

1. What is the anticipated risk of perioperative mortality without intervention?

2. How many participants were included in the studies assessing serious adverse events?

2) Second SoF table

SoF table- Fibrin based haemostatic agents versus non-fibrin-based haemostatic agents

Fibrin-based haemostatic agents compared with non-fibrin-based haemostatic agents reducing intraoperative blood loss and improving outcomes in adult liver surgery

Population: adults undergoing liver resection for cancer or benign disease

Setting: clinical setting in liver resection unit

Intervention: fibrin-based haemostatic agents applied to resection surface

Comparison: non-fibrin-based haemostatic agents applied to resection surface

Outcome	Anticipated risk difference (95% CI)		Relative effect (95% CI)	Number of participants (RCTs)	Certainty of evidence	Comments
	Risk with non-fibrin-based haemostatic agents	Risk with fibrin-based haemostatic agents				
**Perioperative mortality** Median follow-up 1.25 months (range 1 to 3 months)	47 per 1000	48 per 1000	RR 1.03 (0.62 to 1.72)	1436 (11)	⊕ Very low[a,b]	Defined as death, regardless of cause, occurring within 30 days of surgery in or outside hospital
**Serious adverse events**	736 per 1000	729 per 1000 (699 to 758)	RR 0.99 (0.95 to 1.03)	1176 (9)	⊕⊕ Low[a,c]	4 trials defined adverse events according to the Medical Dictionary for Regulatory Activities.
**Reoperation** Median follow-up 1.5 months (range 1 to 3 months)	163 per 1000	78 per 1000 (41 to 147)	RR 0.45 (0.25 to 0.90)	358 (3)	⊕ Very low[a,b]	1 trial reported an exceptionally high rate of reoperation in both groups; this is likely to have skewed the meta-analysis

***The risk in the intervention group** (and its 95% CI) is based on the **assumed risk** in the comparison group and the **relative effect** of the intervention (and its 95% CI).

CI: Confidence interval; **RCT**: Randomised clinical trial; **RR**: risk ratio

GRADE Working Group grades of evidence

High certainty: we are very confident that the true effect lies close to that of the estimate of the effect.

Moderate certainty: we are moderately confident in the effect estimate; the true effect is likely to be close to the estimate of the effect, but there is a possibility that it is substantially different.

Low certainty: our confidence in the effect estimate is limited; the true effect may be substantially different from the estimate of the effect.

Very low certainty: we have very little confidence in the effect estimate; the true effect is likely to be substantially different from the estimate of effect.

[a] – Downgraded one level for study limitations (overall high risk of bias).

[b] – Downgraded two levels for imprecision (few events and wide 95% CI including both benefit and harm).

[c] – Downgraded one level for imprecision (95% CI including both benefit and harm).

1. What is the relative effect of perioperative mortality for patients using fibrin-based haemostatic agents compared to those using non-Fibrin-based haemostatic agents?

2. What is the anticipated risk of serious adverse events in fibrin-based haemostatic agents groups?

3. How many participants were analyzed for serious adverse events?

3) Third SoF table

SoF table- Perampanel add-on versus placebo for drug-resistant focal epilepsy

Perampanel add-on versus placebo for drug-resistant focal epilepsy

Patient or population: People (aged 12 and over) with drug-resistant focal epilepsy

Setting: Outpatients

Intervention: Add-on perampanel (2 mg/ day, 4 mg/ day, 8 mg/ day and 12 mg/ day

Comparison: Add-on placebo

**Outcome**	**Anticipated absolute effects* (95% CI)**		**Relative effect (95% CI)**	**Number of participants (RCT)**	**Certainty of evidence**	**Comments**
	**Risk with placebo**	**Risk with perampanel**				
**> 50% reduction in seizure frequency**<br>Follow-up: 12-19 weeks	205 per 1000	342 per 1000	RR 1.67 (1.43 to 1.95)	2524 (7)	⊕⊕⊕⊕<br>High	Perampanel increases the proportion of participants who achieve a > 50% reduction in seizure frequency.
**Seizure freedom**<br>Follow-up: 19 weeks	18 per 1000	45 per 1000	RR 2.50 (1.38 to 4.54)	2323 (5)	⊕⊕<br>Low[a]	Perampanel may increase the proportion of participants who attain seizure freedom.
**Treatment withdrawal due to any reason**<br>Follow-up: 12-19 weeks	118 per 1000	154 per 1000	RR 1.30 (1.03 to 1.63)	2524 (7)	⊕⊕<br>Low[b,c]	Perampanel may increase the proportion of participants who withdraw from treatment due to any reason.
**Treatment withdrawal due to adverse effects**	37 per 1000	88 per 1000	RR 2.36 (1.59 to 3.51)	2524 (7)	⊕⊕<br>Low[b,c]	Perampanel may increase the prevalence of treatment withdrawal due to adverse effects.

Follow-up: 12-19 weeks						
**Proportion of participants who experienced >1 adverse effect**	662 per 1000	775 per 1000	RR 1.17 (1.10 to 1.24)	2524 (7)	⊕⊕⊕⊕ High	Perampanel increases the incidence of participants reporting > 1 adverse effect.
**Proportion of participants who experienced ataxia**	0 per 1000	34 per 1000	RR 14.32 (1.09 to 188.31)	1098 (2)	⊕⊕ Low[a]	Perampanel may greatly increase the incidence of ataxia.
**Proportion of participants who experienced dizziness**	91 per 1000	260 per 1000	RR 2.87 (1.45 to 5.70)	2524 (7)	⊕⊕ Low[d]	Perampanel may increase the incidence of dizziness.

***The risk in the intervention group** (and its 95% CI) is based on the a**ssumed risk** in the comparison group and the **relative effect** of the intervention (and its 95% CI).

CI: Confidence interval; **RCT**: Randomised clinical trial; **RR:** risk ratio

GRADE Working Group grades of evidence

High certainty: we are very confident that the true effect lies close to that of the estimate of the effect.

Moderate certainty: we are moderately confident in the effect estimate; the true effect is likely to be close to the estimate of the effect, but there is a possibility that it is substantially different.

Low certainty: our confidence in the effect estimate is limited; the true effect may be substantially different from the estimate of the effect.

Very low certainty: we have very little confidence in the effect estimate; the true effect is likely to be substantially different from the estimate of effect.

[a] – Downgraded twice for imprecision. Number of events (fewer than 100) did not suffice the optimal information size.

[b] -Downgraded once for inconsistency. The direction of effect varied across individual trials. While most trials found a negative effect for perampanel compared to placebo, some found no effect, and one found positive effect.

[c] -Downgraded once for imprecision. Number of events (fewer than 400) did not suffice the optimal information size.

[d] -Downgraded twice due to inconsistency. There was statistical heterogeneity across the dana ($p < 0.10$: $I^2 = 75\%$).

1. What is the relative effect of perampanel on achieving a 50% reduction in seizure frequency?

2. How does perampanel affect the likelihood of treatment withdrawal due to adverse effects?

   a) Increases the likelihood
   b) decreases the likelihood
   c) has no effect

3. Which outcome has the largest relative effect when perampanel is used?

4. For the outcome "Proportion of participants who experienced >1 adverse effect", what can be concluded about the effect of Perampanel compared to placebo?

   a) It does not affect the proportion of participants who experienced >1 adverse effect
   b) It increases the proportion of participants who experienced >1 adverse effect
   c) It may increase the proportion of participants who experienced >1 adverse effect
   d) It probably increases the proportion of participants who experienced >1 adverse effect

5. Now, review the comment provided in the table and compare it to your answer to evaluate whether your perspective aligns with the comment. Would you reconsider your response? If yes, explain why.

   a) No
   b) Yes, _______________________

6. For the outcome "Seizure freedom", what can be concluded about the effect of perampanel compared to placebo?

   a) It does not affect the proportion of participants who experienced >1 adverse effect
   b) It increases the proportion of participants who experienced >1 adverse effect
   c) It may increase the proportion of participants who experienced >1 adverse effect
   d) It probably increases the proportion of participants who experienced >1 adverse effect

7. Now, review the comment provided in the table and compare it to your answer to evaluate whether your perspective aligns with the comment. Would you reconsider your response? If yes, explain why.

a) No
b) Yes, ______________________

4) Fourth SoF table

SoF- Combined mechanical and oral antibiotic bowel preparation versus mechanical bowel preparation alone

Combined mechanical and oral antibiotic bowel preparation versus mechanical bowel preparation

Patient or population: Patients undergoing elective colorectal surgery

Setting: Any type of hospital offering elective colorectal recisions. Both single and multicenter studies are included

Intervention: Mechanical and oral antibiotic bowel preparation

Comparison: Mechanical bowel preparation

**Outcome**	**Anticipated absolute effects* (95% CI)**		**Relative effect (95% CI)**	**Number of participants (RCTs)**	**Certainty of evidence**	**Comments**
	**Risk with mechanical bowel preparation**	**Risk with combined mechanical and oral antibiotic bowel preparation**				
**Surgical site infections** Follow-up: 30 days	137 per 1000	77 per 1000	RR 0.56 (0.42 to 0.74)	3917 (16)	⨁⨁⨁ Moderate[a]	Combined mechanical and oral antibiotic bowel preparation probably results in a reduction in surgical site infections.
**Anastomotic leakage** Follow-up: 30 days	44 per 1000	26 per 1000	RR 0.60 (0.36 to 0.99)	2356 (10)	⨁⨁⨁ Moderate[b]	Combined mechanical and oral antibiotic bowel preparation may result in reduction in anastomotic leakage.
**Mortality** Follow-up: 30 days	18 per 1000	16 per 1000	RR 0.87 (0.27 to 2.82)	639 (3)	⨁⨁⨁ Moderate[c]	Combined mechanical and oral antibiotic bowel preparation may result in no difference in mortality.

**Incidence of postoperative ileus**<br>Follow-up: 30 days	49 per 1000	43 per 1000	RR 0.89 (0.59 to 1.32)	2013 (6)	⊕⊕<br>Low[d,e]	Combined mechanical and oral antibiotic bowel preparation may result in no difference in incidence of postoperative ileus.

***The risk in the intervention group** (and its 95% CI) is based on the a**ssumed risk** in the comparison group and the **relative effect** of the intervention (and its 95% CI).

CI: Confidence interval; **RCT**: Randomised clinical trial; **RR:** risk ratio

GRADE Working Group grades of evidence

High certainty: we are very confident that the true effect lies close to that of the estimate of the effect.

Moderate certainty: we are moderately confident in the effect estimate; the true effect is likely to be close to the estimate of the effect, but there is a possibility that it is substantially different.

Low certainty: our confidence in the effect estimate is limited; the true effect may be substantially different from the estimate of the effect.

Very low certainty: we have very little confidence in the effect estimate; the true effect is likely to be substantially different from the estimate of effect.

[a] – The rating was downgraded by one level due to moderate heterogeneity between studies that could not be explained by the subgroup analyses; I^2= 40%

[b] -The rating was downgraded by one level for imprecision. Few events occurred in the included trials (28 in the intervention group and 52 in the control group) and the confidence intervals include both benefits and no effect.

[c] -The rating was downgraded by one level for imprecision. Few events occurred in the included studies (5 in the intervention group and 6 in the control group) and the confidence intervals include considerable benefits and harm.

[d] -The rating was downgraded by one level due to imprecision, as the confidence interval includes considerable benefit and harm.

[e] -The rating was downgraded by one level due to possible publication bias, as small studies reported statistically significant benefits while larger studies showed as much smaller and statistically non-significant effect.

1. What outcome has the highest number of RCTs in their analysis?

2. What is the anticipated absolute effect of postoperative ileus incidence for combined mechanical and oral antibiotic bowel preparation?

3. How does combined mechanical and oral antibiotic preparation affect mortality at 30-day follow-up compared to mechanical bowel preparation alone?

a. Higher mortality
b. Lower mortality
c. No difference in mortality

4. Considering the CI around RR, which of these two interventions in more likely to reduce incidence for anastomotic leakage?

a. Mechanical bowel preparation
b. Combined mechanical and oral antibiotic bowel preparation
c. There is no difference

5. For the outcome "Surgical site infections", what can be concluded about the effect of combined mechanical and oral antibiotic bowel preparation compared to mechanical bowel preparation alone?

a) It results in increase of surgical site infections
b) It may result in of surgical site infections
c) It probably results in increase of surgical site infections
d) It does not have an effect on surgical site infection

6. Now, review the comment provided in the table and compare it to your answer to evaluate whether your perspective aligns with the comment. Would you reconsider your response? If yes, explain why.

a) No
b) Yes, _______________________

7. For the outcome “Anastomotic leakage”, what can be concluded about the effect of combined mechanical and oral bowel preparation compared to mechanical bowel preparation alone?

a) It results in increase of anastomotic leakage
b) It may result in increase of anastomotic leakage
c) It probably results in increase of anastomotic leakage
d) It does not have an effect on anastomotic leakage

8. Now, review the comment provided in the table and compare it to your answer to evaluate whether your perspective aligns with the comment. Would you reconsider your response? If yes, explain why.

a) No
b) Yes, ______________________

Figure S2. Heatmap of total number of readings (clicks) for the first Summary of Findings (SoF) table, for expert group. The colors correspond to the total number of times participants clicked on each cell, from 0 (yellow) to 105 (dark purple)

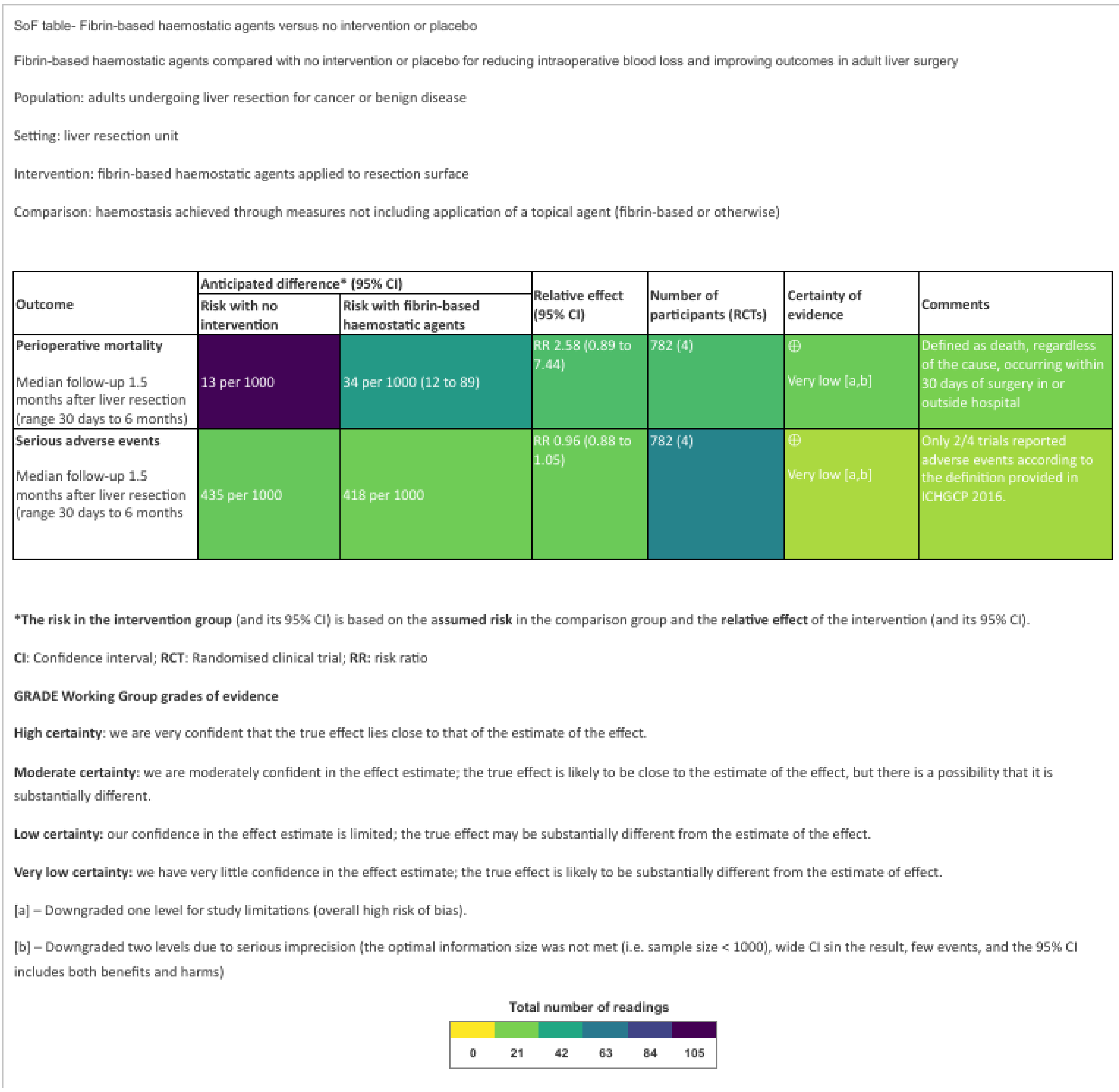

SoF table- Fibrin-based haemostatic agents versus no intervention or placebo

Fibrin-based haemostatic agents compared with no intervention or placebo for reducing intraoperative blood loss and improving outcomes in adult liver surgery

Population: adults undergoing liver resection for cancer or benign disease

Setting: liver resection unit

Intervention: fibrin-based haemostatic agents applied to resection surface

Comparison: haemostasis achieved through measures not including application of a topical agent (fibrin-based or otherwise)

Outcome	Anticipated difference* (95% CI)		Relative effect (95% CI)	Number of participants (RCTs)	Certainty of evidence	Comments
	Risk with no intervention	Risk with fibrin-based haemostatic agents				
**Perioperative mortality**<br><br>Median follow-up 1.5 months after liver resection (range 30 days to 6 months)	13 per 1000	34 per 1000 (12 to 89)	RR 2.58 (0.89 to 7.44)	782 (4)	⊕<br><br>Very low [a,b]	Defined as death, regardless of the cause, occurring within 30 days of surgery in or outside hospital
**Serious adverse events**<br><br>Median follow-up 1.5 months after liver resection (range 30 days to 6 months	435 per 1000	418 per 1000	RR 0.96 (0.88 to 1.05)	782 (4)	⊕<br><br>Very low [a,b]	Only 2/4 trials reported adverse events according to the definition provided in ICHGCP 2016.

***The risk in the intervention group** (and its 95% CI) is based on the **assumed risk** in the comparison group and the **relative effect** of the intervention (and its 95% CI).

CI: Confidence interval; **RCT**: Randomised clinical trial; **RR:** risk ratio

GRADE Working Group grades of evidence

High certainty: we are very confident that the true effect lies close to that of the estimate of the effect.

Moderate certainty: we are moderately confident in the effect estimate; the true effect is likely to be close to the estimate of the effect, but there is a possibility that it is substantially different.

Low certainty: our confidence in the effect estimate is limited; the true effect may be substantially different from the estimate of the effect.

Very low certainty: we have very little confidence in the effect estimate; the true effect is likely to be substantially different from the estimate of effect.

[a] – Downgraded one level for study limitations (overall high risk of bias).

[b] – Downgraded two levels due to serious imprecision (the optimal information size was not met (i.e. sample size < 1000), wide CI sin the result, few events, and the 95% CI includes both benefits and harms)

Total number of readings

0	21	42	63	84	105

Figure S3. Heatmap of total number of reading times (clicks) for the first Summary of Findings (SoF) table, for clinicinas group. The colors correspond to the total number of times participants clicked on each cell, from 0 (yellow) to 105 (dark purple)

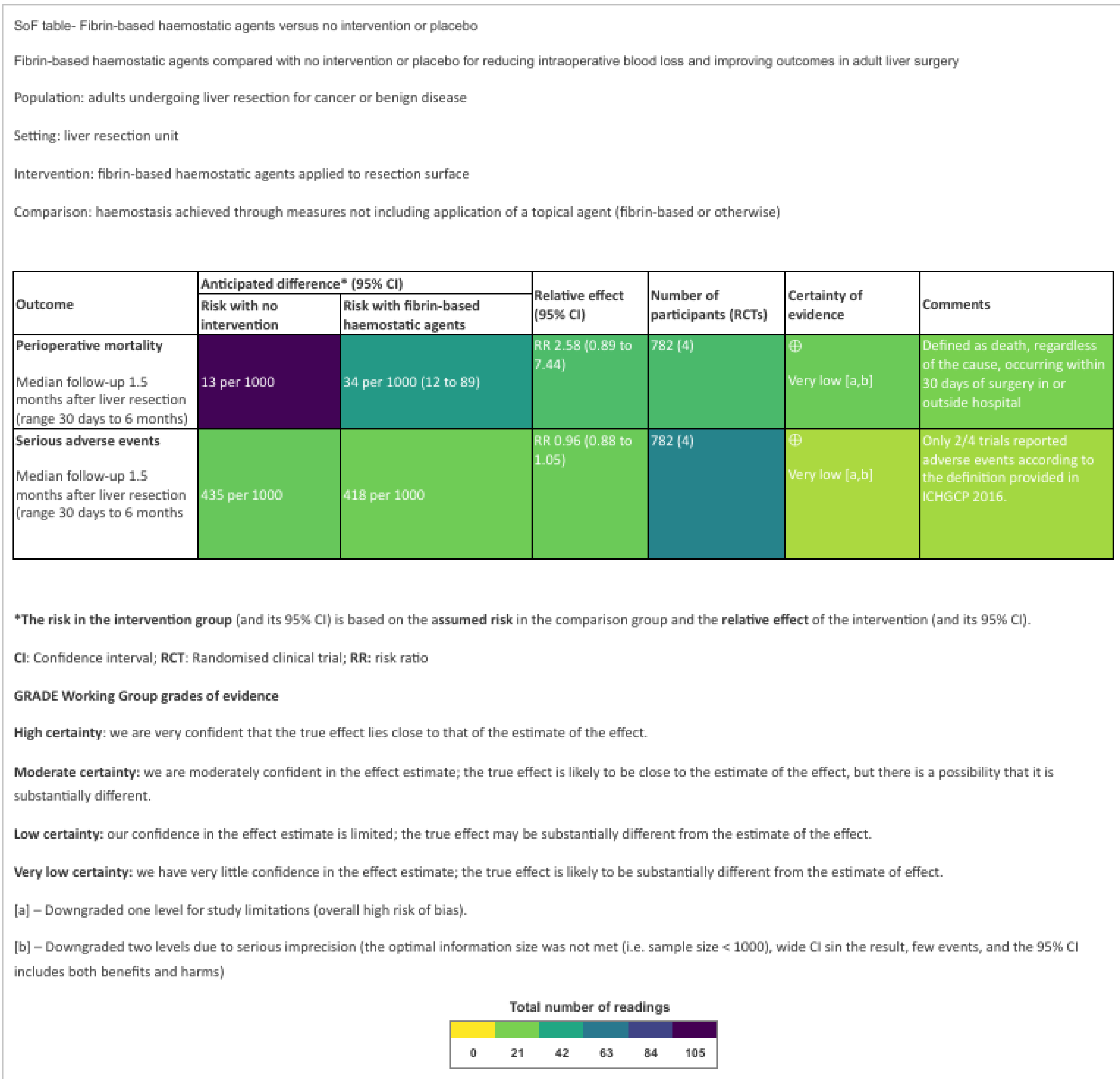

SoF table- Fibrin-based haemostatic agents versus no intervention or placebo

Fibrin-based haemostatic agents compared with no intervention or placebo for reducing intraoperative blood loss and improving outcomes in adult liver surgery

Population: adults undergoing liver resection for cancer or benign disease

Setting: liver resection unit

Intervention: fibrin-based haemostatic agents applied to resection surface

Comparison: haemostasis achieved through measures not including application of a topical agent (fibrin-based or otherwise)

Outcome	Anticipated difference* (95% CI)		Relative effect (95% CI)	Number of participants (RCTs)	Certainty of evidence	Comments
	Risk with no intervention	Risk with fibrin-based haemostatic agents				
**Perioperative mortality**<br><br>Median follow-up 1.5 months after liver resection (range 30 days to 6 months)	13 per 1000	34 per 1000 (12 to 89)	RR 2.58 (0.89 to 7.44)	782 (4)	⊕<br><br>Very low [a,b]	Defined as death, regardless of the cause, occurring within 30 days of surgery in or outside hospital
**Serious adverse events**<br><br>Median follow-up 1.5 months after liver resection (range 30 days to 6 months	435 per 1000	418 per 1000	RR 0.96 (0.88 to 1.05)	782 (4)	⊕<br><br>Very low [a,b]	Only 2/4 trials reported adverse events according to the definition provided in ICHGCP 2016.

***The risk in the intervention group** (and its 95% CI) is based on the **assumed risk** in the comparison group and the **relative effect** of the intervention (and its 95% CI).

CI: Confidence interval; **RCT**: Randomised clinical trial; **RR:** risk ratio

GRADE Working Group grades of evidence

High certainty: we are very confident that the true effect lies close to that of the estimate of the effect.

Moderate certainty: we are moderately confident in the effect estimate; the true effect is likely to be close to the estimate of the effect, but there is a possibility that it is substantially different.

Low certainty: our confidence in the effect estimate is limited; the true effect may be substantially different from the estimate of the effect.

Very low certainty: we have very little confidence in the effect estimate; the true effect is likely to be substantially different from the estimate of effect.

[a] – Downgraded one level for study limitations (overall high risk of bias).

[b] – Downgraded two levels due to serious imprecision (the optimal information size was not met (i.e. sample size < 1000), wide CI sin the result, few events, and the 95% CI includes both benefits and harms)

Figure S4. Heatmap of total number of reading times (clicks) for the first Summary of Findings (SoF) table, for healthcare users group. The colors correspond to the total number of times participants clicked on each cell, from 0 (yellow) to 105 (dark purple)

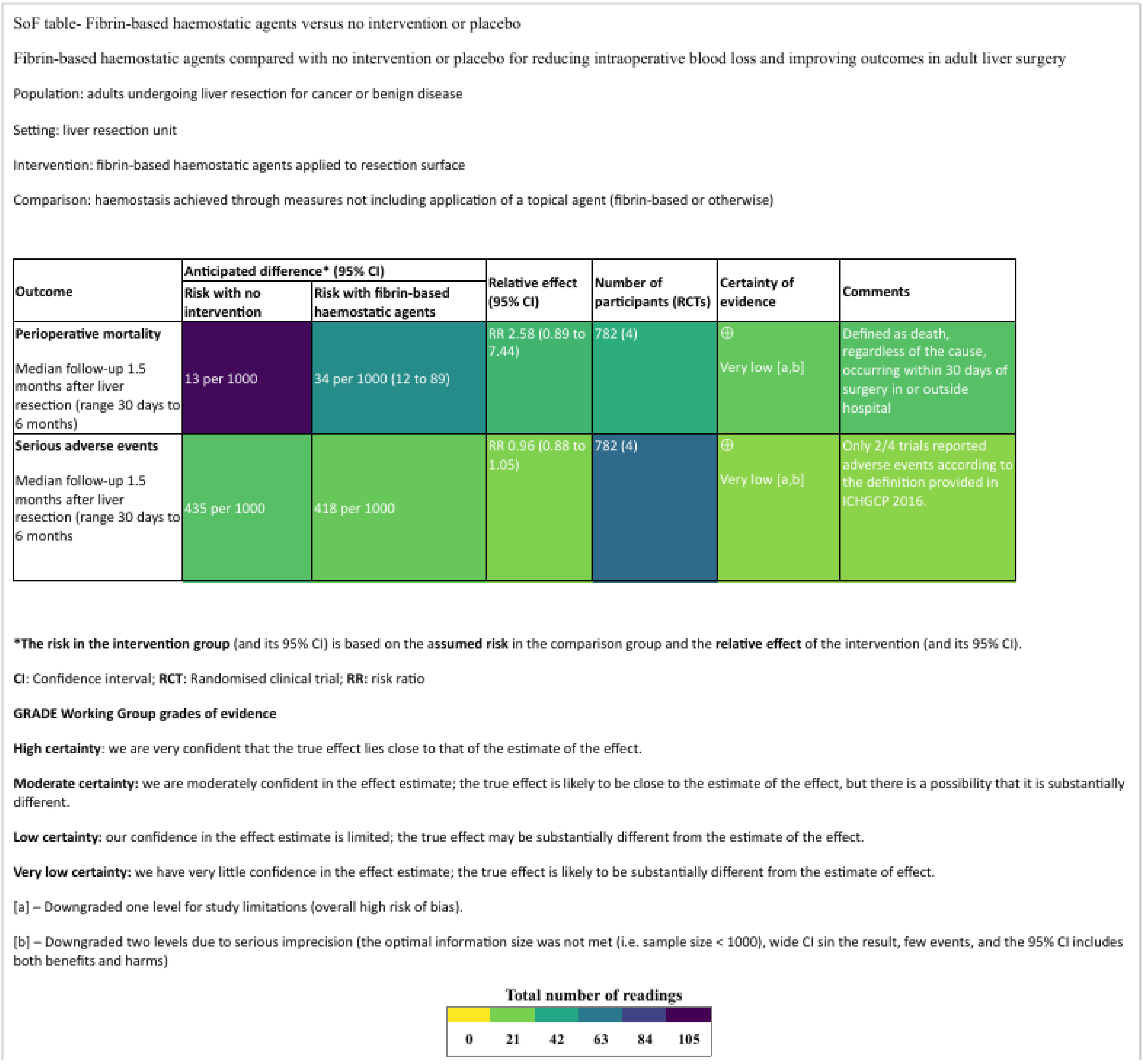

SoF table- Fibrin-based haemostatic agents versus no intervention or placebo

Fibrin-based haemostatic agents compared with no intervention or placebo for reducing intraoperative blood loss and improving outcomes in adult liver surgery

Population: adults undergoing liver resection for cancer or benign disease

Setting: liver resection unit

Intervention: fibrin-based haemostatic agents applied to resection surface

Comparison: haemostasis achieved through measures not including application of a topical agent (fibrin-based or otherwise)

Outcome	Anticipated difference* (95% CI)		Relative effect (95% CI)	Number of participants (RCTs)	Certainty of evidence	Comments
	Risk with no intervention	Risk with fibrin-based haemostatic agents				
**Perioperative mortality**<br><br>Median follow-up 1.5 months after liver resection (range 30 days to 6 months)	13 per 1000	34 per 1000 (12 to 89)	RR 2.58 (0.89 to 7.44)	782 (4)	⊕<br>Very low [a,b]	Defined as death, regardless of the cause, occurring within 30 days of surgery in or outside hospital
**Serious adverse events**<br><br>Median follow-up 1.5 months after liver resection (range 30 days to 6 months	435 per 1000	418 per 1000	RR 0.96 (0.88 to 1.05)	782 (4)	⊕<br>Very low [a,b]	Only 2/4 trials reported adverse events according to the definition provided in ICHGCP 2016.

***The risk in the intervention group** (and its 95% CI) is based on the **assumed risk** in the comparison group and the **relative effect** of the intervention (and its 95% CI).

CI: Confidence interval; **RCT**: Randomised clinical trial; **RR:** risk ratio

GRADE Working Group grades of evidence

High certainty: we are very confident that the true effect lies close to that of the estimate of the effect.

Moderate certainty: we are moderately confident in the effect estimate; the true effect is likely to be close to the estimate of the effect, but there is a possibility that it is substantially different.

Low certainty: our confidence in the effect estimate is limited; the true effect may be substantially different from the estimate of the effect.

Very low certainty: we have very little confidence in the effect estimate; the true effect is likely to be substantially different from the estimate of effect.

[a] – Downgraded one level for study limitations (overall high risk of bias).

[b] – Downgraded two levels due to serious imprecision (the optimal information size was not met (i.e. sample size < 1000), wide CI sin the result, few events, and the 95% CI includes both benefits and harms)

Figure S5. Heatmap of average reading time for the first Summary of Findings (SoF) table for expert group. The colors correspond to the average time spent on the individual table cell, from 0 seconds (yellow) to 15 seconds (dark purple)

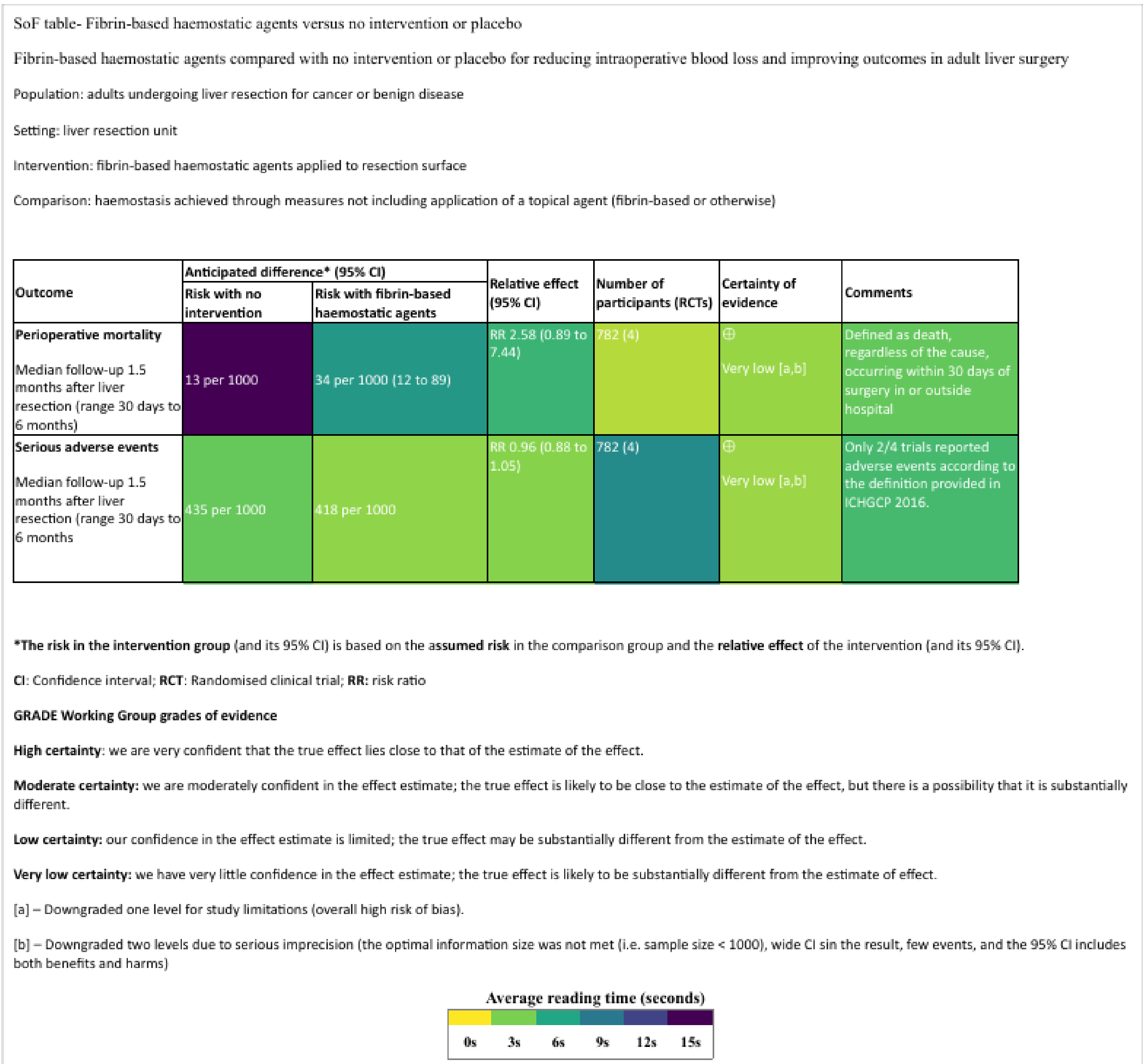

SoF table- Fibrin-based haemostatic agents versus no intervention or placebo

Fibrin-based haemostatic agents compared with no intervention or placebo for reducing intraoperative blood loss and improving outcomes in adult liver surgery

Population: adults undergoing liver resection for cancer or benign disease

Setting: liver resection unit

Intervention: fibrin-based haemostatic agents applied to resection surface

Comparison: haemostasis achieved through measures not including application of a topical agent (fibrin-based or otherwise)

Outcome	Anticipated difference* (95% CI)		Relative effect (95% CI)	Number of participants (RCTs)	Certainty of evidence	Comments
	Risk with no intervention	Risk with fibrin-based haemostatic agents				
**Perioperative mortality**<br><br>Median follow-up 1.5 months after liver resection (range 30 days to 6 months)	13 per 1000	34 per 1000 (12 to 89)	RR 2.58 (0.89 to 7.44)	782 (4)	⊕<br>Very low [a,b]	Defined as death, regardless of the cause, occurring within 30 days of surgery in or outside hospital
**Serious adverse events**<br><br>Median follow-up 1.5 months after liver resection (range 30 days to 6 months	435 per 1000	418 per 1000	RR 0.96 (0.88 to 1.05)	782 (4)	⊕<br>Very low [a,b]	Only 2/4 trials reported adverse events according to the definition provided in ICHGCP 2016.

***The risk in the intervention group** (and its 95% CI) is based on the **assumed risk** in the comparison group and the **relative effect** of the intervention (and its 95% CI).

CI: Confidence interval; **RCT**: Randomised clinical trial; **RR:** risk ratio

GRADE Working Group grades of evidence

High certainty: we are very confident that the true effect lies close to that of the estimate of the effect.

Moderate certainty: we are moderately confident in the effect estimate; the true effect is likely to be close to the estimate of the effect, but there is a possibility that it is substantially different.

Low certainty: our confidence in the effect estimate is limited; the true effect may be substantially different from the estimate of the effect.

Very low certainty: we have very little confidence in the effect estimate; the true effect is likely to be substantially different from the estimate of effect.

[a] – Downgraded one level for study limitations (overall high risk of bias).

[b] – Downgraded two levels due to serious imprecision (the optimal information size was not met (i.e. sample size < 1000), wide CI sin the result, few events, and the 95% CI includes both benefits and harms)

Figure S6. Heatmap of average reading time for the first Summary of Findings (SoF) table for clinician group. The colors correspond to the average time spent on the individual table cell, from 0 seconds (yellow) to 15 seconds (dark purple)

SoF table- Fibrin-based haemostatic agents versus no intervention or placebo

Fibrin-based haemostatic agents compared with no intervention or placebo for reducing intraoperative blood loss and improving outcomes in adult liver surgery

Population: adults undergoing liver resection for cancer or benign disease

Setting: liver resection unit

Intervention: fibrin-based haemostatic agents applied to resection surface

Comparison: haemostasis achieved through measures not including application of a topical agent (fibrin-based or otherwise)

Outcome	Anticipated difference* (95% CI)		Relative effect (95% CI)	Number of participants (RCTs)	Certainty of evidence	Comments
	Risk with no intervention	Risk with fibrin-based haemostatic agents				
**Perioperative mortality**<br><br>Median follow-up 1.5 months after liver resection (range 30 days to 6 months)	13 per 1000	34 per 1000 (12 to 89)	RR 2.58 (0.89 to 7.44)	782 (4)	⊕<br><br>Very low [a,b]	Defined as death, regardless of the cause, occurring within 30 days of surgery in or outside hospital
**Serious adverse events**<br><br>Median follow-up 1.5 months after liver resection (range 30 days to 6 months	435 per 1000	418 per 1000	RR 0.96 (0.88 to 1.05)	782 (4)	⊕<br><br>Very low [a,b]	Only 2/4 trials reported adverse events according to the definition provided in ICHGCP 2016.

***The risk in the intervention group** (and its 95% CI) is based on the **assumed risk** in the comparison group and the **relative effect** of the intervention (and its 95% CI).

CI: Confidence interval; **RCT**: Randomised clinical trial; **RR:** risk ratio

GRADE Working Group grades of evidence

High certainty: we are very confident that the true effect lies close to that of the estimate of the effect.

Moderate certainty: we are moderately confident in the effect estimate; the true effect is likely to be close to the estimate of the effect, but there is a possibility that it is substantially different.

Low certainty: our confidence in the effect estimate is limited; the true effect may be substantially different from the estimate of the effect.

Very low certainty: we have very little confidence in the effect estimate; the true effect is likely to be substantially different from the estimate of effect.

[a] – Downgraded one level for study limitations (overall high risk of bias).

[b] – Downgraded two levels due to serious imprecision (the optimal information size was not met (i.e. sample size < 1000), wide CI sin the result, few events, and the 95% CI includes both benefits and harms)

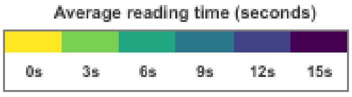

Figure S7. Heatmap of average reading time for the first Summary of Findings (SoF) table for healthcare user group. The colors correspond to the average time spent on the individual table cell, from 0 seconds (yellow) to 15 seconds (dark purple)

SoF table- Fibrin-based haemostatic agents versus no intervention or placebo

Fibrin-based haemostatic agents compared with no intervention or placebo for reducing intraoperative blood loss and improving outcomes in adult liver surgery

Population: adults undergoing liver resection for cancer or benign disease

Setting: liver resection unit

Intervention: fibrin-based haemostatic agents applied to resection surface

Comparison: haemostasis achieved through measures not including application of a topical agent (fibrin-based or otherwise)

Outcome	Anticipated difference* (95% CI)		Relative effect (95% CI)	Number of participants (RCTs)	Certainty of evidence	Comments
	Risk with no intervention	Risk with fibrin-based haemostatic agents				
**Perioperative mortality**<br><br>Median follow-up 1.5 months after liver resection (range 30 days to 6 months)	13 per 1000	34 per 1000 (12 to 89)	RR 2.58 (0.89 to 7.44)	782 (4)	⊕<br><br>Very low [a,b]	Defined as death, regardless of the cause, occurring within 30 days of surgery in or outside hospital
**Serious adverse events**<br><br>Median follow-up 1.5 months after liver resection (range 30 days to 6 months	435 per 1000	418 per 1000	RR 0.96 (0.88 to 1.05)	782 (4)	⊕<br><br>Very low [a,b]	Only 2/4 trials reported adverse events according to the definition provided in ICHGCP 2016.

***The risk in the intervention group** (and its 95% CI) is based on the **assumed risk** in the comparison group and the **relative effect** of the intervention (and its 95% CI).

CI: Confidence interval; **RCT**: Randomised clinical trial; **RR:** risk ratio

GRADE Working Group grades of evidence

High certainty: we are very confident that the true effect lies close to that of the estimate of the effect.

Moderate certainty: we are moderately confident in the effect estimate; the true effect is likely to be close to the estimate of the effect, but there is a possibility that it is substantially different.

Low certainty: our confidence in the effect estimate is limited; the true effect may be substantially different from the estimate of the effect.

Very low certainty: we have very little confidence in the effect estimate; the true effect is likely to be substantially different from the estimate of effect.

[a] – Downgraded one level for study limitations (overall high risk of bias).

[b] – Downgraded two levels due to serious imprecision (the optimal information size was not met (i.e. sample size < 1000), wide CI sin the result, few events, and the 95% CI includes both benefits and harms)

Average reading time (seconds)

0s 3s 6s 9s 12s 15s

Figure S8. Heatmap of total number of reading times (clicks) for the second Summary of Findings (SoF) table, for the expert group. The colors correspond to the total number of times participants clicked on each cell, from 0 (yellow) to 105 (dark purple)

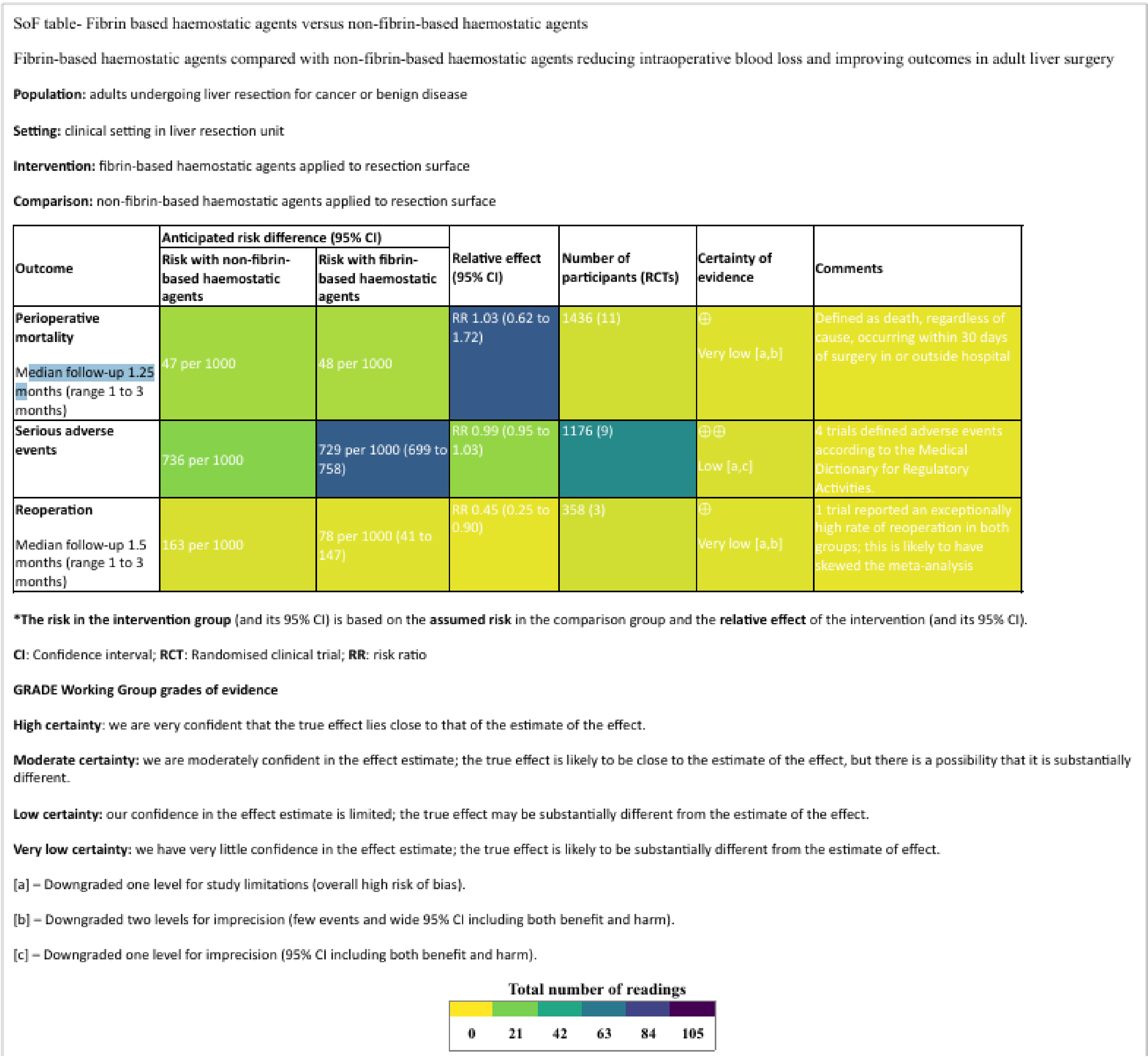

SoF table- Fibrin based haemostatic agents versus non-fibrin-based haemostatic agents

Fibrin-based haemostatic agents compared with non-fibrin-based haemostatic agents reducing intraoperative blood loss and improving outcomes in adult liver surgery

Population: adults undergoing liver resection for cancer or benign disease

Setting: clinical setting in liver resection unit

Intervention: fibrin-based haemostatic agents applied to resection surface

Comparison: non-fibrin-based haemostatic agents applied to resection surface

Outcome	Anticipated risk difference (95% CI)		Relative effect (95% CI)	Number of participants (RCTs)	Certainty of evidence	Comments
	Risk with non-fibrin-based haemostatic agents	Risk with fibrin-based haemostatic agents				
**Perioperative mortality**<br><br>Median follow-up 1.25 months (range 1 to 3 months)	47 per 1000	48 per 1000	RR 1.03 (0.62 to 1.72)	1436 (11)	⊕<br><br>Very low [a,b]	Defined as death, regardless of cause, occurring within 30 days of surgery in or outside hospital
**Serious adverse events**	736 per 1000	729 per 1000 (699 to 758)	RR 0.99 (0.95 to 1.03)	1176 (9)	⊕⊕<br><br>Low [a,c]	4 trials defined adverse events according to the Medical Dictionary for Regulatory Activities.
**Reoperation**<br><br>Median follow-up 1.5 months (range 1 to 3 months)	163 per 1000	78 per 1000 (41 to 147)	RR 0.45 (0.25 to 0.90)	358 (3)	⊕<br><br>Very low [a,b]	1 trial reported an exceptionally high rate of reoperation in both groups; this is likely to have skewed the meta-analysis

***The risk in the intervention group** (and its 95% CI) is based on the **assumed risk** in the comparison group and the **relative effect** of the intervention (and its 95% CI).

CI: Confidence interval; **RCT**: Randomised clinical trial; **RR**: risk ratio

GRADE Working Group grades of evidence

High certainty: we are very confident that the true effect lies close to that of the estimate of the effect.

Moderate certainty: we are moderately confident in the effect estimate; the true effect is likely to be close to the estimate of the effect, but there is a possibility that it is substantially different.

Low certainty: our confidence in the effect estimate is limited; the true effect may be substantially different from the estimate of the effect.

Very low certainty: we have very little confidence in the effect estimate; the true effect is likely to be substantially different from the estimate of effect.

[a] – Downgraded one level for study limitations (overall high risk of bias).

[b] – Downgraded two levels for imprecision (few events and wide 95% CI including both benefit and harm).

[c] – Downgraded one level for imprecision (95% CI including both benefit and harm).

Figure S9. Heatmap of total number of reading times (clicks) for the second Summary of Findings (SoF) table, for the clinician group. The colors correspond to the total number of times participants clicked on each cell, from 0 (yellow) to 105 (dark purple)

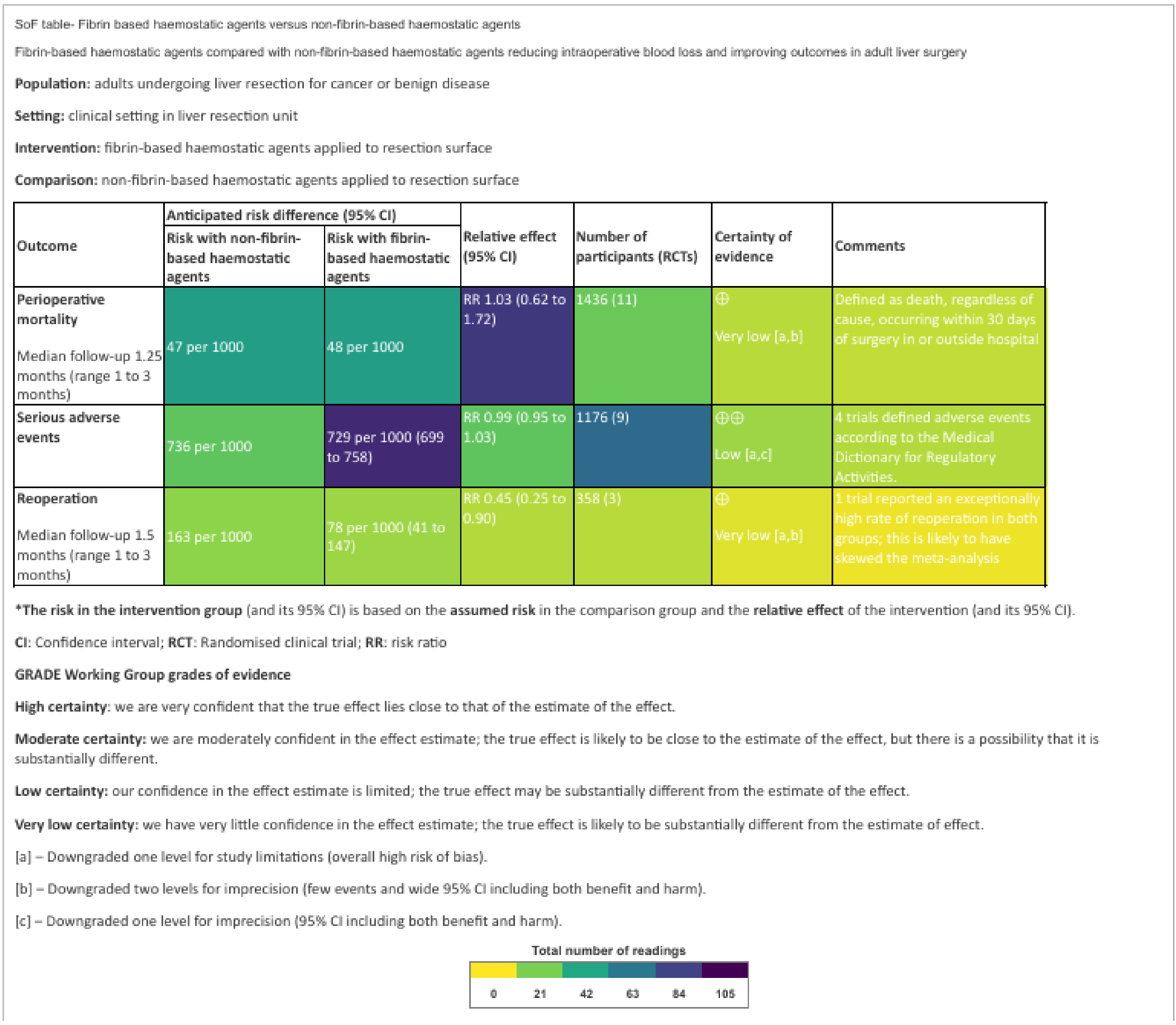

SoF table- Fibrin based haemostatic agents versus non-fibrin-based haemostatic agents

Fibrin-based haemostatic agents compared with non-fibrin-based haemostatic agents reducing intraoperative blood loss and improving outcomes in adult liver surgery

Population: adults undergoing liver resection for cancer or benign disease

Setting: clinical setting in liver resection unit

Intervention: fibrin-based haemostatic agents applied to resection surface

Comparison: non-fibrin-based haemostatic agents applied to resection surface

Outcome	Anticipated risk difference (95% CI)		Relative effect (95% CI)	Number of participants (RCTs)	Certainty of evidence	Comments
	Risk with non-fibrin-based haemostatic agents	Risk with fibrin-based haemostatic agents				
Perioperative mortality<br><br>Median follow-up 1.25 months (range 1 to 3 months)	47 per 1000	48 per 1000	RR 1.03 (0.62 to 1.72)	1436 (11)	⊕<br>Very low [a,b]	Defined as death, regardless of cause, occurring within 30 days of surgery in or outside hospital
Serious adverse events	736 per 1000	729 per 1000 (699 to 758)	RR 0.99 (0.95 to 1.03)	1176 (9)	⊕⊕<br>Low [a,c]	4 trials defined adverse events according to the Medical Dictionary for Regulatory Activities.
Reoperation<br><br>Median follow-up 1.5 months (range 1 to 3 months)	163 per 1000	78 per 1000 (41 to 147)	RR 0.45 (0.25 to 0.90)	358 (3)	⊕<br>Very low [a,b]	1 trial reported an exceptionally high rate of reoperation in both groups; this is likely to have skewed the meta-analysis

***The risk in the intervention group** (and its 95% CI) is based on the **assumed risk** in the comparison group and the **relative effect** of the intervention (and its 95% CI).

CI: Confidence interval; **RCT**: Randomised clinical trial; **RR**: risk ratio

GRADE Working Group grades of evidence

High certainty: we are very confident that the true effect lies close to that of the estimate of the effect.

Moderate certainty: we are moderately confident in the effect estimate; the true effect is likely to be close to the estimate of the effect, but there is a possibility that it is substantially different.

Low certainty: our confidence in the effect estimate is limited; the true effect may be substantially different from the estimate of the effect.

Very low certainty: we have very little confidence in the effect estimate; the true effect is likely to be substantially different from the estimate of effect.

[a] – Downgraded one level for study limitations (overall high risk of bias).

[b] – Downgraded two levels for imprecision (few events and wide 95% CI including both benefit and harm).

[c] – Downgraded one level for imprecision (95% CI including both benefit and harm).

Figure S10. Heatmap of total number of reading times (clicks) for the second Summary of Findings (SoF) table, for the healthcare user group. The colors correspond to the total number of times participants clicked on each cell, from 0 (yellow) to 105 (dark purple)

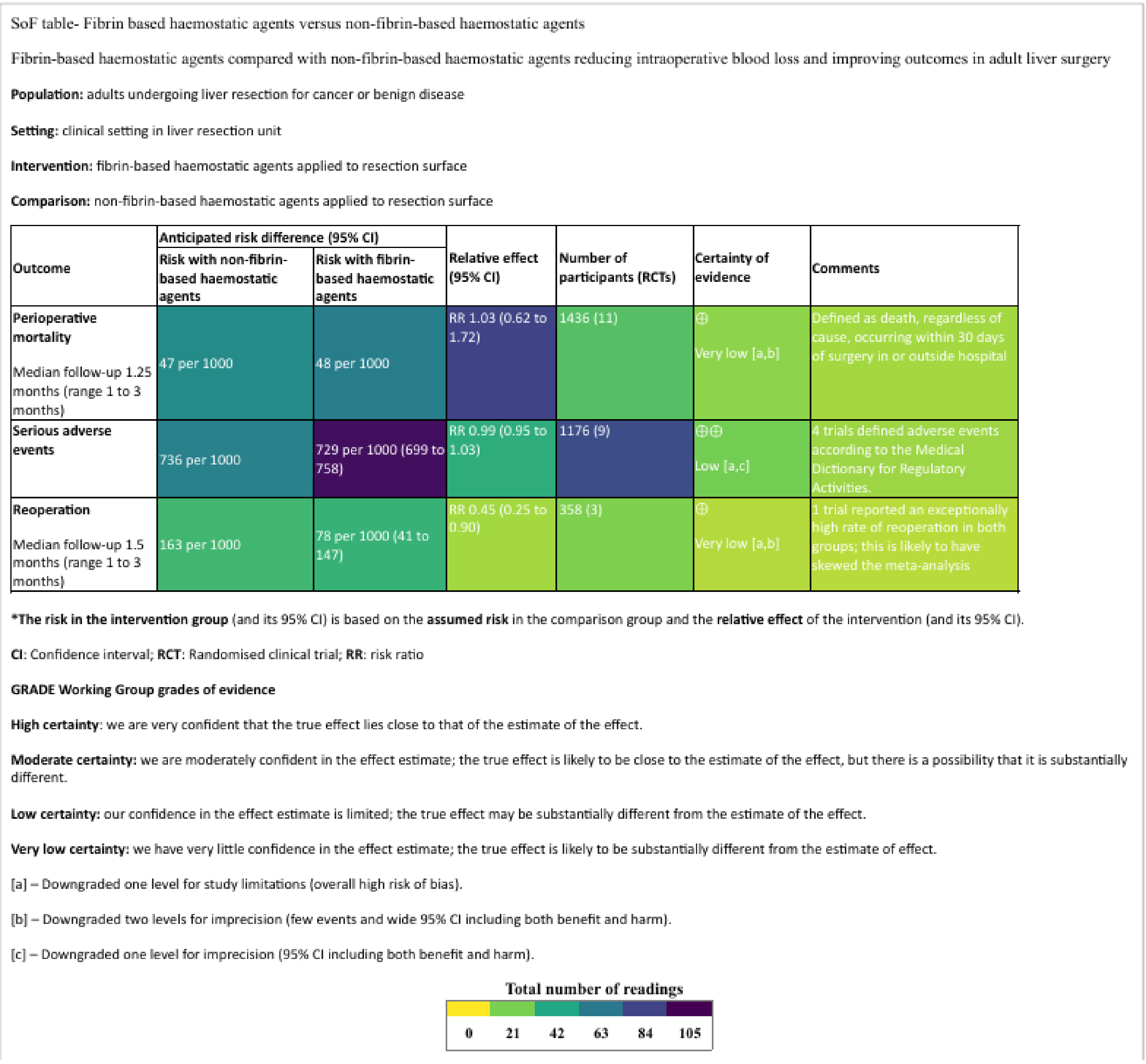

SoF table- Fibrin based haemostatic agents versus non-fibrin-based haemostatic agents

Fibrin-based haemostatic agents compared with non-fibrin-based haemostatic agents reducing intraoperative blood loss and improving outcomes in adult liver surgery

Population: adults undergoing liver resection for cancer or benign disease

Setting: clinical setting in liver resection unit

Intervention: fibrin-based haemostatic agents applied to resection surface

Comparison: non-fibrin-based haemostatic agents applied to resection surface

Outcome	Anticipated risk difference (95% CI)		Relative effect (95% CI)	Number of participants (RCTs)	Certainty of evidence	Comments
	Risk with non-fibrin-based haemostatic agents	Risk with fibrin-based haemostatic agents				
**Perioperative mortality** Median follow-up 1.25 months (range 1 to 3 months)	47 per 1000	48 per 1000	RR 1.03 (0.62 to 1.72)	1436 (11)	⊕ Very low [a,b]	Defined as death, regardless of cause, occurring within 30 days of surgery in or outside hospital
**Serious adverse events**	736 per 1000	729 per 1000 (699 to 758)	RR 0.99 (0.95 to 1.03)	1176 (9)	⊕⊕ Low [a,c]	4 trials defined adverse events according to the Medical Dictionary for Regulatory Activities.
**Reoperation** Median follow-up 1.5 months (range 1 to 3 months)	163 per 1000	78 per 1000 (41 to 147)	RR 0.45 (0.25 to 0.90)	358 (3)	⊕ Very low [a,b]	1 trial reported an exceptionally high rate of reoperation in both groups; this is likely to have skewed the meta-analysis

***The risk in the intervention group** (and its 95% CI) is based on the **assumed risk** in the comparison group and the **relative effect** of the intervention (and its 95% CI).

CI: Confidence interval; **RCT**: Randomised clinical trial; **RR**: risk ratio

GRADE Working Group grades of evidence

High certainty: we are very confident that the true effect lies close to that of the estimate of the effect.

Moderate certainty: we are moderately confident in the effect estimate; the true effect is likely to be close to the estimate of the effect, but there is a possibility that it is substantially different.

Low certainty: our confidence in the effect estimate is limited; the true effect may be substantially different from the estimate of the effect.

Very low certainty: we have very little confidence in the effect estimate; the true effect is likely to be substantially different from the estimate of effect.

[a] – Downgraded one level for study limitations (overall high risk of bias).

[b] – Downgraded two levels for imprecision (few events and wide 95% CI including both benefit and harm).

[c] – Downgraded one level for imprecision (95% CI including both benefit and harm).

Figure S11. Heatmap of average reading time for the second Summary of Findings (SoF) table for the expert group. The colors correspond to the average time spent on the individual table cell, from 0 seconds (yellow) to 15 seconds (dark purple)

SoF table- Fibrin based haemostatic agents versus non-fibrin-based haemostatic agents

Fibrin-based haemostatic agents compared with non-fibrin-based haemostatic agents reducing intraoperative blood loss and improving outcomes in adult liver surgery

Population: adults undergoing liver resection for cancer or benign disease

Setting: clinical setting in liver resection unit

Intervention: fibrin-based haemostatic agents applied to resection surface

Comparison: non-fibrin-based haemostatic agents applied to resection surface

Outcome	Anticipated risk difference (95% CI)		Relative effect (95% CI)	Number of participants (RCTs)	Certainty of evidence	Comments
	Risk with non-fibrin-based haemostatic agents	Risk with fibrin-based haemostatic agents				
**Perioperative mortality**<br><br>Median follow-up 1.25 months (range 1 to 3 months)	47 per 1000	48 per 1000	RR 1.03 (0.62 to 1.72)	1436 (11)	⊕<br><br>Very low [a,b]	Defined as death, regardless of cause, occurring within 30 days of surgery in or outside hospital
**Serious adverse events**	736 per 1000	729 per 1000 (699 to 758)	RR 0.99 (0.95 to 1.03)	1176 (9)	⊕⊕<br><br>Low [a,c]	4 trials defined adverse events according to the Medical Dictionary for Regulatory Activities.
**Reoperation**<br><br>Median follow-up 1.5 months (range 1 to 3 months)	163 per 1000	78 per 1000 (41 to 147)	RR 0.45 (0.25 to 0.90)	358 (3)	⊕<br><br>Very low [a,b]	1 trial reported an exceptionally high rate of reoperation in both groups; this is likely to have skewed the meta-analysis

***The risk in the intervention group** (and its 95% CI) is based on the **assumed risk** in the comparison group and the **relative effect** of the intervention (and its 95% CI).

CI: Confidence interval; **RCT**: Randomised clinical trial; **RR**: risk ratio

GRADE Working Group grades of evidence

High certainty: we are very confident that the true effect lies close to that of the estimate of the effect.

Moderate certainty: we are moderately confident in the effect estimate; the true effect is likely to be close to the estimate of the effect, but there is a possibility that it is substantially different.

Low certainty: our confidence in the effect estimate is limited; the true effect may be substantially different from the estimate of the effect.

Very low certainty: we have very little confidence in the effect estimate; the true effect is likely to be substantially different from the estimate of effect.

[a] – Downgraded one level for study limitations (overall high risk of bias).

[b] – Downgraded two levels for imprecision (few events and wide 95% CI including both benefit and harm).

[c] – Downgraded one level for imprecision (95% CI including both benefit and harm).

Average reading time (seconds)

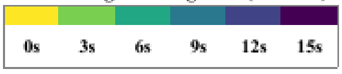

Figure S12. Heatmap of average reading time for the second Summary of Findings (SoF) table for the clinician group. The colors correspond to the average time spent on the individual table cell, from 0 seconds (yellow) to 15 seconds (dark purple)

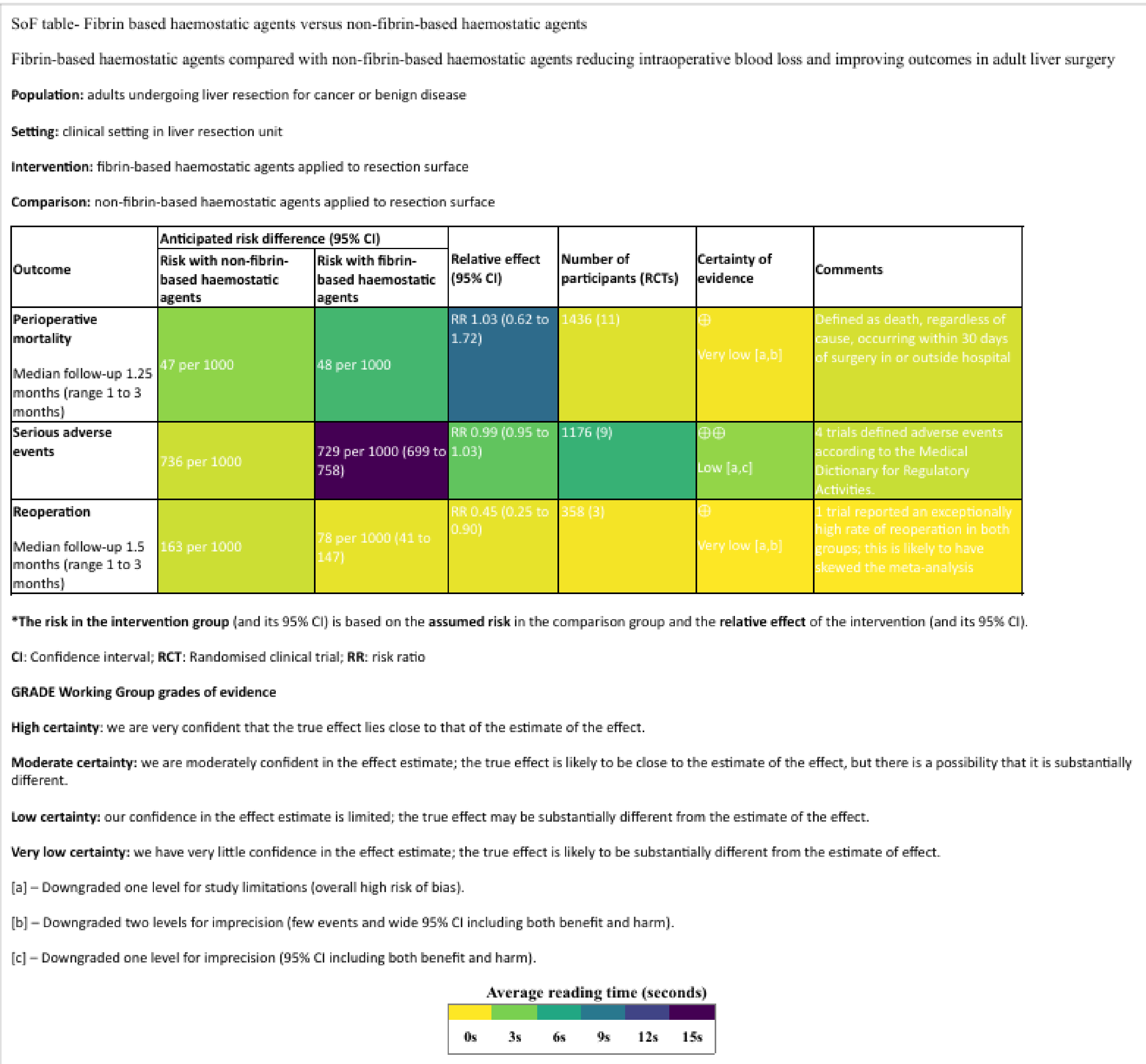

SoF table- Fibrin based haemostatic agents versus non-fibrin-based haemostatic agents

Fibrin-based haemostatic agents compared with non-fibrin-based haemostatic agents reducing intraoperative blood loss and improving outcomes in adult liver surgery

Population: adults undergoing liver resection for cancer or benign disease

Setting: clinical setting in liver resection unit

Intervention: fibrin-based haemostatic agents applied to resection surface

Comparison: non-fibrin-based haemostatic agents applied to resection surface

Outcome	Anticipated risk difference (95% CI)		Relative effect (95% CI)	Number of participants (RCTs)	Certainty of evidence	Comments
	Risk with non-fibrin-based haemostatic agents	Risk with fibrin-based haemostatic agents				
**Perioperative mortality**<br>Median follow-up 1.25 months (range 1 to 3 months)	47 per 1000	48 per 1000	RR 1.03 (0.62 to 1.72)	1436 (11)	⊕<br>Very low [a,b]	Defined as death, regardless of cause, occurring within 30 days of surgery in or outside hospital
**Serious adverse events**	736 per 1000	729 per 1000 (699 to 758)	RR 0.99 (0.95 to 1.03)	1176 (9)	⊕⊕<br>Low [a,c]	4 trials defined adverse events according to the Medical Dictionary for Regulatory Activities.
**Reoperation**<br>Median follow-up 1.5 months (range 1 to 3 months)	163 per 1000	78 per 1000 (41 to 147)	RR 0.45 (0.25 to 0.90)	358 (3)	⊕<br>Very low [a,b]	1 trial reported an exceptionally high rate of reoperation in both groups; this is likely to have skewed the meta-analysis

***The risk in the intervention group** (and its 95% CI) is based on the **assumed risk** in the comparison group and the **relative effect** of the intervention (and its 95% CI).

CI: Confidence interval; **RCT**: Randomised clinical trial; **RR**: risk ratio

GRADE Working Group grades of evidence

High certainty: we are very confident that the true effect lies close to that of the estimate of the effect.

Moderate certainty: we are moderately confident in the effect estimate; the true effect is likely to be close to the estimate of the effect, but there is a possibility that it is substantially different.

Low certainty: our confidence in the effect estimate is limited; the true effect may be substantially different from the estimate of the effect.

Very low certainty: we have very little confidence in the effect estimate; the true effect is likely to be substantially different from the estimate of effect.

[a] – Downgraded one level for study limitations (overall high risk of bias).

[b] – Downgraded two levels for imprecision (few events and wide 95% CI including both benefit and harm).

[c] – Downgraded one level for imprecision (95% CI including both benefit and harm).

Figure S13. Heatmap of average reading time for the second Summary of Findings (SoF) table for the healthcare user group. The colors correspond to the average time spent on the individual table cell, from 0 seconds (yellow) to 15 seconds (dark purple)

SoF table- Fibrin based haemostatic agents versus non-fibrin-based haemostatic agents

Fibrin-based haemostatic agents compared with non-fibrin-based haemostatic agents reducing intraoperative blood loss and improving outcomes in adult liver surgery

Population: adults undergoing liver resection for cancer or benign disease

Setting: clinical setting in liver resection unit

Intervention: fibrin-based haemostatic agents applied to resection surface

Comparison: non-fibrin-based haemostatic agents applied to resection surface

Outcome	Anticipated risk difference (95% CI)		Relative effect (95% CI)	Number of participants (RCTs)	Certainty of evidence	Comments
	Risk with non-fibrin-based haemostatic agents	Risk with fibrin-based haemostatic agents				
**Perioperative mortality**<br><br>Median follow-up 1.25 months (range 1 to 3 months)	47 per 1000	48 per 1000	RR 1.03 (0.62 to 1.72)	1436 (11)	⊕<br><br>Very low [a,b]	Defined as death, regardless of cause, occurring within 30 days of surgery in or outside hospital
**Serious adverse events**	736 per 1000	729 per 1000 (699 to 758)	RR 0.99 (0.95 to 1.03)	1176 (9)	⊕⊕<br><br>Low [a,c]	4 trials defined adverse events according to the Medical Dictionary for Regulatory Activities.
**Reoperation**<br><br>Median follow-up 1.5 months (range 1 to 3 months)	163 per 1000	78 per 1000 (41 to 147)	RR 0.45 (0.25 to 0.90)	358 (3)	⊕<br><br>Very low [a,b]	1 trial reported an exceptionally high rate of reoperation in both groups; this is likely to have skewed the meta-analysis

***The risk in the intervention group** (and its 95% CI) is based on the **assumed risk** in the comparison group and the **relative effect** of the intervention (and its 95% CI).

CI: Confidence interval; **RCT**: Randomised clinical trial; **RR**: risk ratio

GRADE Working Group grades of evidence

High certainty: we are very confident that the true effect lies close to that of the estimate of the effect.

Moderate certainty: we are moderately confident in the effect estimate; the true effect is likely to be close to the estimate of the effect, but there is a possibility that it is substantially different.

Low certainty: our confidence in the effect estimate is limited; the true effect may be substantially different from the estimate of the effect.

Very low certainty: we have very little confidence in the effect estimate; the true effect is likely to be substantially different from the estimate of effect.

[a] – Downgraded one level for study limitations (overall high risk of bias).

[b] – Downgraded two levels for imprecision (few events and wide 95% CI including both benefit and harm).

[c] – Downgraded one level for imprecision (95% CI including both benefit and harm).

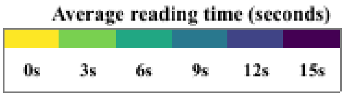

Figure S14. Heatmap of total number of reading times (clicks) for the third Summary of Findings (SoF) table, for the experts groups. The colors correspond to the total number of times participants clicked on each cell, from 0 (yellow) to 105 (dark purple)

SoF table- Perampanel add-on versus placebo for drug-resistant focal epilepsy

Perampanel add-on versus placebo for drug-resistant focal epilepsy

Patient or population: People (aged 12 and over) with drug-resistant focal epilepsy

Setting: Outpatients

Intervention: Add-on perampanel (2 mg/ day, 4 mg/ day, 8 mg/ day and 12 mg/ day

Comparison: Add-on placebo

**Outcome**	**Anticipated absolute effects* (95% CI)**		**Relative effect (95% CI)**	**Number of participants (RCT)**	**Certainty of evidence**	**Comments**
	**Risk with placebo**	**Risk with perampanel**				
**> 50% reduction in seizure frequency**<br>Follow-up: 12-19 weeks	205 per 1000	342 per 1000	RR 1.67 (1.43 to 1.95)	2524 (7)	⊕⊕⊕⊕<br>High	Perampanel increases the proportion of participants who achieve a > 50% reduction in seizure frequency.
**Seizure freedom**<br>Follow-up: 19 weeks	18 per 1000	45 per 1000	RR 2.50 (1.38 to 4.54)	2323 (5)	⊕⊕<br>Low [a]	Perampanel may increase the proportion of participants who attain seizure freedom.
**Treatment withdrawal due to any reason**<br>Follow-up: 12-19 weeks	118 per 1000	154 per 1000	RR 1.30 (1.03 to 1.63)	2524 (7)	⊕⊕<br>Low [b,c]	Perampanel may increase the proportion of participants who withdraw from treatment due to any reason.
**Treatment withdrawal due to adverse effects**<br>Follow-up: 12-19 weeks	37 per 1000	88 per 1000	RR 2.36 (1.59 to 3.51)	2524 (7)	⊕⊕<br>Low [b,c]	Perampanel may increase the prevalence of treatment withdrawal due to adverse effects.
**Proportion of participants who experienced >1 adverse effect**	662 per 1000	775 per 1000	RR 1.17 (1.10 to 1.24)	2524 (7)	⊕⊕⊕⊕<br>High	Perampanel increases the incidence of participants reporting > 1 adverse effect.
**Proportion of participants who experienced ataxia**	0 per 1000	34 per 1000	RR 14.32 (1.09 to 188.31)	1098 (2)	⊕⊕<br>Low [a]	Perampanel may greatly increase the incidence of ataxia.
**Proportion of participants who experienced dizziness**	91 per 1000	260 per 1000	RR 2.87 (1.45 to 5.70)	2524 (7)	⊕⊕<br>Low [d]	Perampanel may increase the incidence of dizziness.

***The risk in the intervention group** (and its 95% CI) is based on the **assumed risk** in the comparison group and the **relative effect** of the intervention (and its 95% CI).

CI: Confidence interval; **RCT:** Randomised clinical trial; **RR:** risk ratio

GRADE Working Group grades of evidence

High certainty: we are very confident that the true effect lies close to that of the estimate of the effect.

Moderate certainty: we are moderately confident in the effect estimate; the true effect is likely to be close to the estimate of the effect, but there is a possibility that it is substantially different.

Low certainty: our confidence in the effect estimate is limited; the true effect may be substantially different from the estimate of the effect.

Very low certainty: we have very little confidence in the effect estimate; the true effect is likely to be substantially different from the estimate of effect.

[a] – Downgraded twice for imprecision. Number of events (fewer than 100) did not suffice the optimal information size.

[b] -Downgraded once for inconsistency. The direction of effect varied across individual trials. While most trials found a negative effect for perampanel compared to placebo, some found no effect, and one found positive effect.

[c] -Downgraded once for imprecision. Number of events (fewer than 400) did not suffice the optimal information size.

[d] -Downgraded twice due to inconsistency. There was statistical heterogeneity across the data (p <0.10: I2= 75%).

Total number of readings

0	21	42	63	84	105

Figure S15. Heatmap of total number of reading times (clicks) for the third Summary of Findings (SoF) table, for the clinician groups. The colors correspond to the total number of times participants clicked on each cell, from 0 (yellow) to 105 (dark purple)

SoF table- Perampanel add-on versus placebo for drug-resistant focal epilepsy

Perampanel add-on versus placebo for drug-resistant focal epilepsy

Patient or population: People (aged 12 and over) with drug-resistant focal epilepsy

Setting: Outpatients

Intervention: Add-on perampanel (2 mg/ day, 4 mg/ day, 8 mg/ day and 12 mg/ day

Comparison: Add-on placebo

**Outcome**	**Anticipated absolute effects* (95% CI)**		**Relative effect (95% CI)**	**Number of participants (RCT)**	**Certainty of evidence**	**Comments**
	**Risk with placebo**	**Risk with perampanel**				
**> 50% reduction in seizure frequency**<br>Follow-up: 12-19 weeks	205 per 1000	342 per 1000	RR 1.67 (1.43 to 1.95)	2524 (7)	⊕⊕⊕⊕<br>High	Perampanel increases the proportion of participants who achieve a > 50% reduction in seizure frequency.
**Seizure freedom**<br>Follow-up: 19 weeks	18 per 1000	45 per 1000	RR 2.50 (1.38 to 4.54)	2323 (5)	⊕⊕<br>Low [a]	Perampanel may increase the proportion of participants who attain seizure freedom.
**Treatment withdrawal due to any reason**<br>Follow-up: 12-19 weeks	118 per 1000	154 per 1000	RR 1.30 (1.03 to 1.63)	2524 (7)	⊕⊕<br>Low [b,c]	Perampanel may increase the proportion of participants who withdraw from treatment due to any reason.
**Treatment withdrawal due to adverse effects**<br>Follow-up: 12-19 weeks	37 per 1000	88 per 1000	RR 2.36 (1.59 to 3.51)	2524 (7)	⊕⊕<br>Low [b,c]	Perampanel may increase the prevalence of treatment withdrawal due to adverse effects.
**Proportion of participants who experienced >1 adverse effect**	662 per 1000	775 per 1000	RR 1.17 (1.10 to 1.24)	2524 (7)	⊕⊕⊕⊕<br>High	Perampanel increases the incidence of participants reporting > 1 adverse effect.
**Proportion of participants who experienced ataxia**	0 per 1000	34 per 1000	RR 14.32 (1.09 to 188.31)	1098 (2)	⊕⊕<br>Low [a]	Perampanel may greatly increase the incidence of ataxia.
**Proportion of participants who experienced dizziness**	91 per 1000	260 per 1000	RR 2.87 (1.45 to 5.70)	2524 (7)	⊕⊕<br>Low [d]	Perampanel may increase the incidence of dizziness.

***The risk in the intervention group** (and its 95% CI) is based on the **assumed risk** in the comparison group and the **relative effect** of the intervention (and its 95% CI).

CI: Confidence interval; **RCT**: Randomised clinical trial; **RR:** risk ratio

GRADE Working Group grades of evidence

High certainty: we are very confident that the true effect lies close to that of the estimate of the effect.

Moderate certainty: we are moderately confident in the effect estimate; the true effect is likely to be close to the estimate of the effect, but there is a possibility that it is substantially different.

Low certainty: our confidence in the effect estimate is limited; the true effect may be substantially different from the estimate of the effect.

Very low certainty: we have very little confidence in the effect estimate; the true effect is likely to be substantially different from the estimate of effect.

[a] – Downgraded twice for imprecision. Number of events (fewer than 100) did not suffice the optimal information size.

[b] -Downgraded once for inconsistency. The direction of effect varied across individual trials. While most trials found a negative effect for perampanel compared to placebo, some found no effect, and one found positive effect.

[c] -Downgraded once for imprecision. Number of events (fewer than 400) did not suffice the optimal information size.

[d] -Downgraded twice due to inconsistency. There was statistical heterogeneity across the data (p <0.10: I2= 75%).

Total number of readings

0	21	42	63	84	105

Figure S16. Heatmap of total number of reading times (clicks) for the third Summary of Findings (SoF) table, for the healthcare user groups. The colors correspond to the total number of times participants clicked on each cell, from 0 (yellow) to 105 (dark purple)

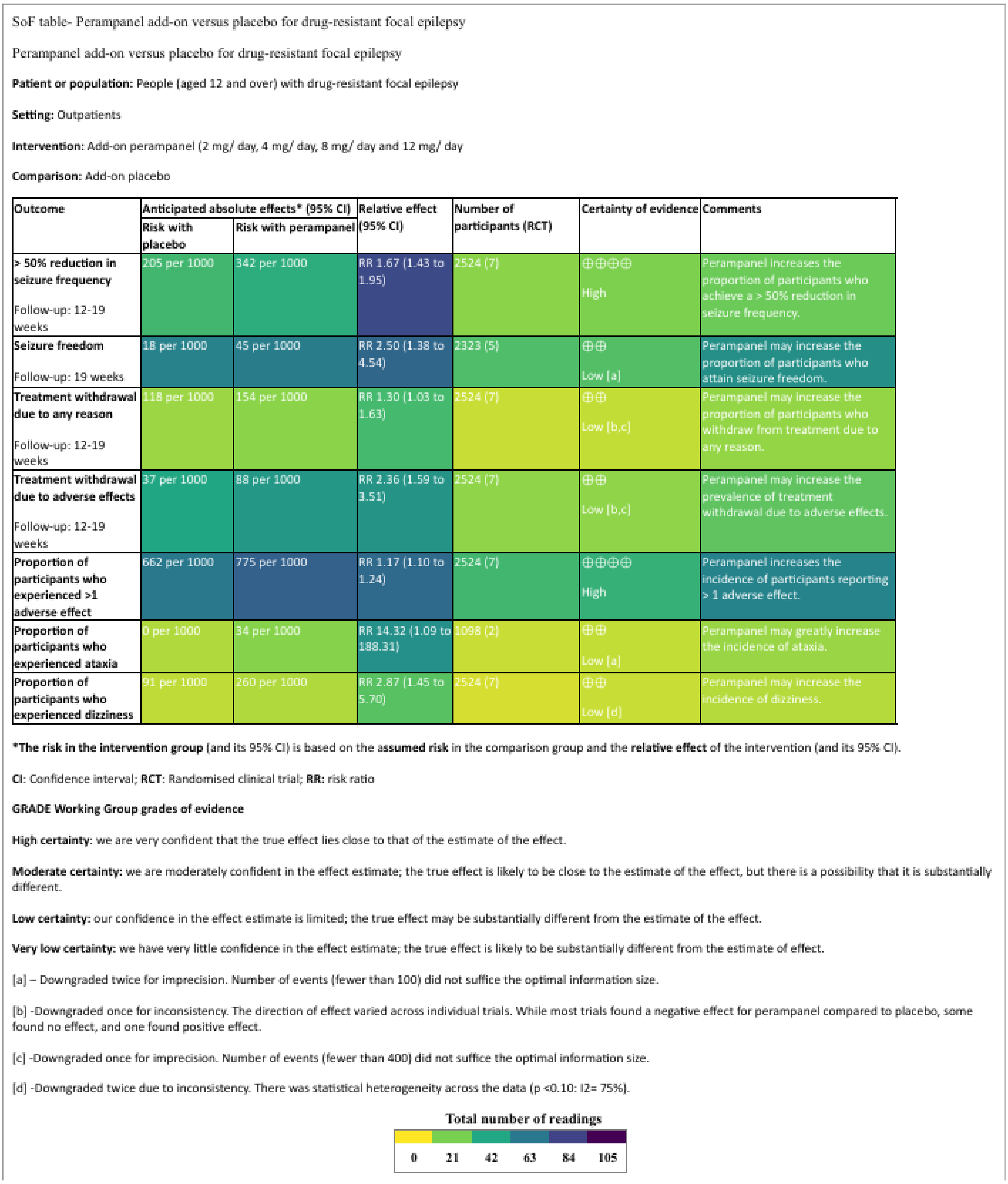

SoF table- Perampanel add-on versus placebo for drug-resistant focal epilepsy

Perampanel add-on versus placebo for drug-resistant focal epilepsy

Patient or population: People (aged 12 and over) with drug-resistant focal epilepsy

Setting: Outpatients

Intervention: Add-on perampanel (2 mg/ day, 4 mg/ day, 8 mg/ day and 12 mg/ day

Comparison: Add-on placebo

Outcome	Anticipated absolute effects* (95% CI)		Relative effect (95% CI)	Number of participants (RCT)	Certainty of evidence	Comments
	Risk with placebo	Risk with perampanel				
> 50% reduction in seizure frequency<br>Follow-up: 12-19 weeks	205 per 1000	342 per 1000	RR 1.67 (1.43 to 1.95)	2524 (7)	⊕⊕⊕⊕<br>High	Perampanel increases the proportion of participants who achieve a > 50% reduction in seizure frequency.
Seizure freedom<br>Follow-up: 19 weeks	18 per 1000	45 per 1000	RR 2.50 (1.38 to 4.54)	2323 (5)	⊕⊕<br>Low [a]	Perampanel may increase the proportion of participants who attain seizure freedom.
Treatment withdrawal due to any reason<br>Follow-up: 12-19 weeks	118 per 1000	154 per 1000	RR 1.30 (1.03 to 1.63)	2524 (7)	⊕⊕<br>Low [b,c]	Perampanel may increase the proportion of participants who withdraw from treatment due to any reason.
Treatment withdrawal due to adverse effects<br>Follow-up: 12-19 weeks	37 per 1000	88 per 1000	RR 2.36 (1.59 to 3.51)	2524 (7)	⊕⊕<br>Low [b,c]	Perampanel may increase the prevalence of treatment withdrawal due to adverse effects.
Proportion of participants who experienced >1 adverse effect	662 per 1000	775 per 1000	RR 1.17 (1.10 to 1.24)	2524 (7)	⊕⊕⊕⊕<br>High	Perampanel increases the incidence of participants reporting > 1 adverse effect.
Proportion of participants who experienced ataxia	0 per 1000	34 per 1000	RR 14.32 (1.09 to 188.31)	1098 (2)	⊕⊕<br>Low [a]	Perampanel may greatly increase the incidence of ataxia.
Proportion of participants who experienced dizziness	91 per 1000	260 per 1000	RR 2.87 (1.45 to 5.70)	2524 (7)	⊕⊕<br>Low [d]	Perampanel may increase the incidence of dizziness.

***The risk in the intervention group** (and its 95% CI) is based on the **assumed risk** in the comparison group and the **relative effect** of the intervention (and its 95% CI).

CI: Confidence interval; **RCT**: Randomised clinical trial; **RR**: risk ratio

GRADE Working Group grades of evidence

High certainty: we are very confident that the true effect lies close to that of the estimate of the effect.

Moderate certainty: we are moderately confident in the effect estimate; the true effect is likely to be close to the estimate of the effect, but there is a possibility that it is substantially different.

Low certainty: our confidence in the effect estimate is limited; the true effect may be substantially different from the estimate of the effect.

Very low certainty: we have very little confidence in the effect estimate; the true effect is likely to be substantially different from the estimate of effect.

[a] – Downgraded twice for imprecision. Number of events (fewer than 100) did not suffice the optimal information size.

[b] -Downgraded once for inconsistency. The direction of effect varied across individual trials. While most trials found a negative effect for perampanel compared to placebo, some found no effect, and one found positive effect.

[c] -Downgraded once for imprecision. Number of events (fewer than 400) did not suffice the optimal information size.

[d] -Downgraded twice due to inconsistency. There was statistical heterogeneity across the data (p <0.10: I2= 75%).

Figure S17. Heatmap of average reading time for the third Summary of Findings (SoF) table for the expert groups. The colors correspond to the average time spent on the individual table cell, from 0 seconds (yellow) to 15 seconds (dark purple)

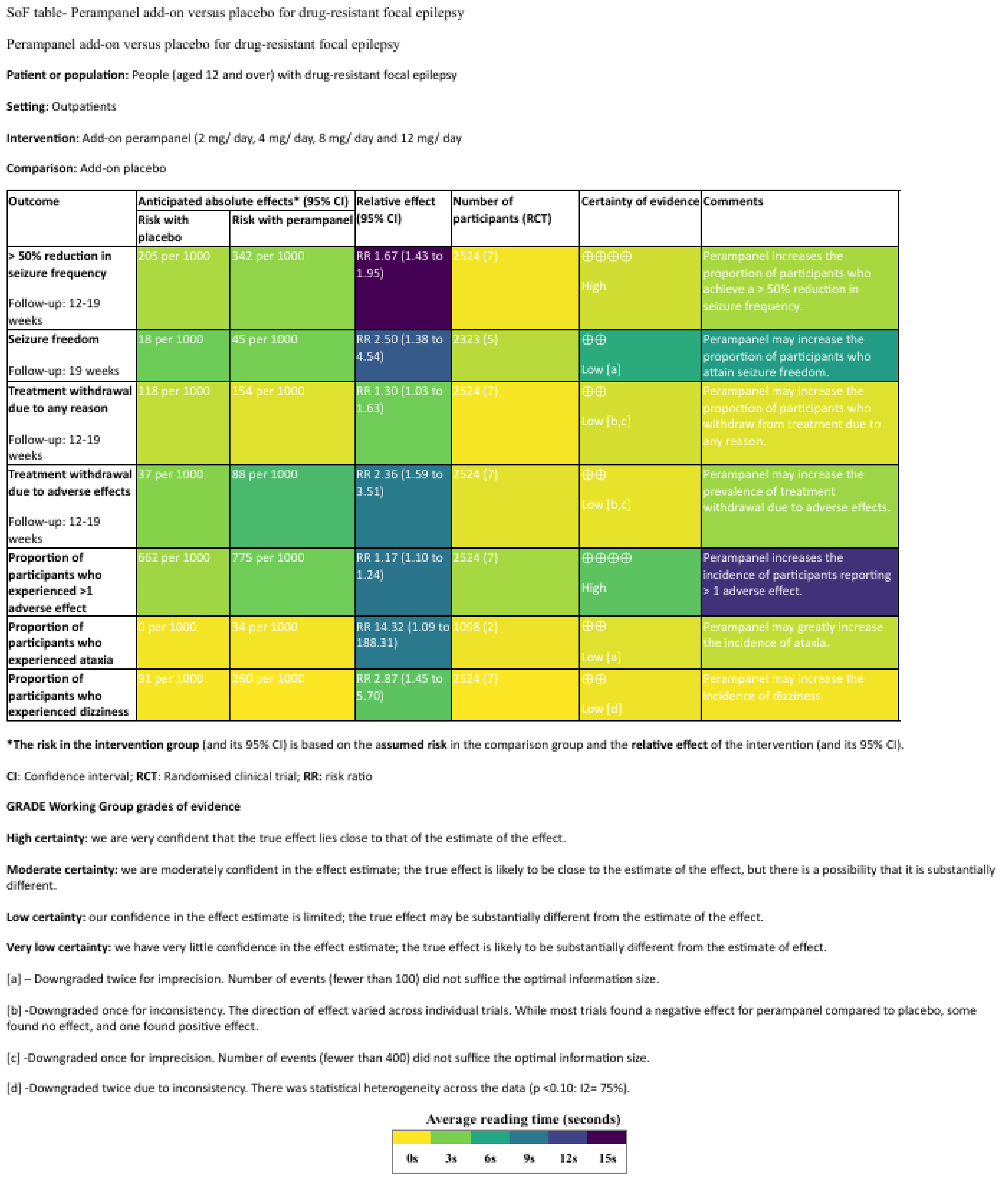

SoF table- Perampanel add-on versus placebo for drug-resistant focal epilepsy

Perampanel add-on versus placebo for drug-resistant focal epilepsy

Patient or population: People (aged 12 and over) with drug-resistant focal epilepsy

Setting: Outpatients

Intervention: Add-on perampanel (2 mg/ day, 4 mg/ day, 8 mg/ day and 12 mg/ day

Comparison: Add-on placebo

Outcome	Anticipated absolute effects* (95% CI)		Relative effect (95% CI)	Number of participants (RCT)	Certainty of evidence	Comments
	Risk with placebo	Risk with perampanel				
> 50% reduction in seizure frequency<br>Follow-up: 12-19 weeks	205 per 1000	342 per 1000	RR 1.67 (1.43 to 1.95)	2524 (7)	⊕⊕⊕⊕<br>High	Perampanel increases the proportion of participants who achieve a > 50% reduction in seizure frequency.
Seizure freedom<br>Follow-up: 19 weeks	18 per 1000	45 per 1000	RR 2.50 (1.38 to 4.54)	2323 (5)	⊕⊕<br>Low [a]	Perampanel may increase the proportion of participants who attain seizure freedom.
Treatment withdrawal due to any reason<br>Follow-up: 12-19 weeks	118 per 1000	154 per 1000	RR 1.30 (1.03 to 1.63)	2524 (7)	⊕⊕<br>Low [b,c]	Perampanel may increase the proportion of participants who withdraw from treatment due to any reason.
Treatment withdrawal due to adverse effects<br>Follow-up: 12-19 weeks	37 per 1000	88 per 1000	RR 2.36 (1.59 to 3.51)	2524 (7)	⊕⊕<br>Low [b,c]	Perampanel may increase the prevalence of treatment withdrawal due to adverse effects.
Proportion of participants who experienced >1 adverse effect	662 per 1000	775 per 1000	RR 1.17 (1.10 to 1.24)	2524 (7)	⊕⊕⊕⊕<br>High	Perampanel increases the incidence of participants reporting > 1 adverse effect.
Proportion of participants who experienced ataxia	0 per 1000	34 per 1000	RR 14.32 (1.09 to 188.31)	1098 (2)	⊕⊕<br>Low [a]	Perampanel may greatly increase the incidence of ataxia.
Proportion of participants who experienced dizziness	91 per 1000	260 per 1000	RR 2.87 (1.45 to 5.70)	2524 (7)	⊕⊕<br>Low [d]	Perampanel may increase the incidence of dizziness.

***The risk in the intervention group** (and its 95% CI) is based on the **assumed risk** in the comparison group and the **relative effect** of the intervention (and its 95% CI).

CI: Confidence interval; **RCT**: Randomised clinical trial; **RR:** risk ratio

GRADE Working Group grades of evidence

High certainty: we are very confident that the true effect lies close to that of the estimate of the effect.

Moderate certainty: we are moderately confident in the effect estimate; the true effect is likely to be close to the estimate of the effect, but there is a possibility that it is substantially different.

Low certainty: our confidence in the effect estimate is limited; the true effect may be substantially different from the estimate of the effect.

Very low certainty: we have very little confidence in the effect estimate; the true effect is likely to be substantially different from the estimate of effect.

[a] – Downgraded twice for imprecision. Number of events (fewer than 100) did not suffice the optimal information size.

[b] -Downgraded once for inconsistency. The direction of effect varied across individual trials. While most trials found a negative effect for perampanel compared to placebo, some found no effect, and one found positive effect.

[c] -Downgraded once for imprecision. Number of events (fewer than 400) did not suffice the optimal information size.

[d] -Downgraded twice due to inconsistency. There was statistical heterogeneity across the data (p <0.10: I2= 75%).

Figure S18. Heatmap of average reading time for the third Summary of Findings (SoF) table for the clinicnas group. The colors correspond to the average time spent on the individual table cell, from 0 seconds (yellow) to 15 seconds (dark purple)

SoF table- Perampanel add-on versus placebo for drug-resistant focal epilepsy

Perampanel add-on versus placebo for drug-resistant focal epilepsy

Patient or population: People (aged 12 and over) with drug-resistant focal epilepsy

Setting: Outpatients

Intervention: Add-on perampanel (2 mg/ day, 4 mg/ day, 8 mg/ day and 12 mg/ day

Comparison: Add-on placebo

Outcome	Anticipated absolute effects* (95% CI)		Relative effect (95% CI)	Number of participants (RCT)	Certainty of evidence	Comments
	Risk with placebo	Risk with perampanel				
> 50% reduction in seizure frequency<br>Follow-up: 12-19 weeks	205 per 1000	342 per 1000	RR 1.67 (1.43 to 1.95)	2524 (7)	⊕⊕⊕⊕<br>High	Perampanel increases the proportion of participants who achieve a > 50% reduction in seizure frequency.
Seizure freedom<br>Follow-up: 19 weeks	18 per 1000	45 per 1000	RR 2.50 (1.38 to 4.54)	2323 (5)	⊕⊕<br>Low [a]	Perampanel may increase the proportion of participants who attain seizure freedom.
Treatment withdrawal due to any reason<br>Follow-up: 12-19 weeks	118 per 1000	154 per 1000	RR 1.30 (1.03 to 1.63)	2524 (7)	⊕⊕<br>Low [b,c]	Perampanel may increase the proportion of participants who withdraw from treatment due to any reason.
Treatment withdrawal due to adverse effects<br>Follow-up: 12-19 weeks	37 per 1000	88 per 1000	RR 2.36 (1.59 to 3.51)	2524 (7)	⊕⊕<br>Low [b,c]	Perampanel may increase the prevalence of treatment withdrawal due to adverse effects.
Proportion of participants who experienced >1 adverse effect	662 per 1000	775 per 1000	RR 1.17 (1.10 to 1.24)	2524 (7)	⊕⊕⊕⊕<br>High	Perampanel increases the incidence of participants reporting > 1 adverse effect.
Proportion of participants who experienced ataxia	0 per 1000	34 per 1000	RR 14.32 (1.09 to 188.31)	1098 (2)	⊕⊕<br>Low [a]	Perampanel may greatly increase the incidence of ataxia.
Proportion of participants who experienced dizziness	91 per 1000	260 per 1000	RR 2.87 (1.45 to 5.70)	2524 (7)	⊕⊕<br>Low [d]	Perampanel may increase the incidence of dizziness.

***The risk in the intervention group** (and its 95% CI) is based on the **assumed risk** in the comparison group and the **relative effect** of the intervention (and its 95% CI).

CI: Confidence interval; **RCT**: Randomised clinical trial; **RR**: risk ratio

GRADE Working Group grades of evidence

High certainty: we are very confident that the true effect lies close to that of the estimate of the effect.

Moderate certainty: we are moderately confident in the effect estimate; the true effect is likely to be close to the estimate of the effect, but there is a possibility that it is substantially different.

Low certainty: our confidence in the effect estimate is limited; the true effect may be substantially different from the estimate of the effect.

Very low certainty: we have very little confidence in the effect estimate; the true effect is likely to be substantially different from the estimate of effect.

[a] – Downgraded twice for imprecision. Number of events (fewer than 100) did not suffice the optimal information size.

[b] -Downgraded once for inconsistency. The direction of effect varied across individual trials. While most trials found a negative effect for perampanel compared to placebo, some found no effect, and one found positive effect.

[c] -Downgraded once for imprecision. Number of events (fewer than 400) did not suffice the optimal information size.

[d] -Downgraded twice due to inconsistency. There was statistical heterogeneity across the data (p <0.10: I2= 75%).

Average reading time (seconds)

0s 3s 6s 9s 12s 15s

Figure S19. Heatmap of average reading time for the third Summary of Findings (SoF) table for the healthcare user group. The colors correspond to the average time spent on the individual table cell, from 0 seconds (yellow) to 15 seconds (dark purple)

SoF table- Perampanel add-on versus placebo for drug-resistant focal epilepsy

Perampanel add-on versus placebo for drug-resistant focal epilepsy

Patient or population: People (aged 12 and over) with drug-resistant focal epilepsy

Setting: Outpatients

Intervention: Add-on perampanel (2 mg/ day, 4 mg/ day, 8 mg/ day and 12 mg/ day

Comparison: Add-on placebo

Outcome	Anticipated absolute effects* (95% CI)		Relative effect (95% CI)	Number of participants (RCT)	Certainty of evidence	Comments
	Risk with placebo	Risk with perampanel				
> 50% reduction in seizure frequency<br><br>Follow-up: 12-19 weeks	205 per 1000	342 per 1000	RR 1.67 (1.43 to 1.95)	2524 (7)	⊕⊕⊕⊕<br>High	Perampanel increases the proportion of participants who achieve a > 50% reduction in seizure frequency.
Seizure freedom<br><br>Follow-up: 19 weeks	18 per 1000	45 per 1000	RR 2.50 (1.38 to 4.54)	2323 (5)	⊕⊕<br>Low [a]	Perampanel may increase the proportion of participants who attain seizure freedom.
Treatment withdrawal due to any reason<br><br>Follow-up: 12-19 weeks	118 per 1000	154 per 1000	RR 1.30 (1.03 to 1.63)	2524 (7)	⊕⊕<br>Low [b,c]	Perampanel may increase the proportion of participants who withdraw from treatment due to any reason.
Treatment withdrawal due to adverse effects<br><br>Follow-up: 12-19 weeks	37 per 1000	88 per 1000	RR 2.36 (1.59 to 3.51)	2524 (7)	⊕⊕<br>Low [b,c]	Perampanel may increase the prevalence of treatment withdrawal due to adverse effects.
Proportion of participants who experienced >1 adverse effect	662 per 1000	775 per 1000	RR 1.17 (1.10 to 1.24)	2524 (7)	⊕⊕⊕⊕<br>High	Perampanel increases the incidence of participants reporting > 1 adverse effect.
Proportion of participants who experienced ataxia	0 per 1000	34 per 1000	RR 14.32 (1.09 to 188.31)	1098 (2)	⊕⊕<br>Low [a]	Perampanel may greatly increase the incidence of ataxia.
Proportion of participants who experienced dizziness	91 per 1000	260 per 1000	RR 2.87 (1.45 to 5.70)	2524 (7)	⊕⊕<br>Low [d]	Perampanel may increase the incidence of dizziness.

***The risk in the intervention group** (and its 95% CI) is based on the **assumed risk** in the comparison group and the **relative effect** of the intervention (and its 95% CI).

CI: Confidence interval; **RCT:** Randomised clinical trial; **RR:** risk ratio

GRADE Working Group grades of evidence

High certainty: we are very confident that the true effect lies close to that of the estimate of the effect.

Moderate certainty: we are moderately confident in the effect estimate; the true effect is likely to be close to the estimate of the effect, but there is a possibility that it is substantially different.

Low certainty: our confidence in the effect estimate is limited; the true effect may be substantially different from the estimate of the effect.

Very low certainty: we have very little confidence in the effect estimate; the true effect is likely to be substantially different from the estimate of effect.

[a] – Downgraded twice for imprecision. Number of events (fewer than 100) did not suffice the optimal information size.

[b] -Downgraded once for inconsistency. The direction of effect varied across individual trials. While most trials found a negative effect for perampanel compared to placebo, some found no effect, and one found positive effect.

[c] -Downgraded once for imprecision. Number of events (fewer than 400) did not suffice the optimal information size.

[d] -Downgraded twice due to inconsistency. There was statistical heterogeneity across the data (p <0.10: I2= 75%).

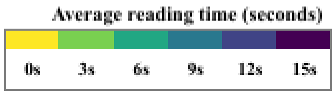

Table S1. Distribution of medical specialties among clinicians (n = 40)

Medical specialty	Frequency (n=40)	% of Total
Dental medicine	6	15.0
Psychiatry	5	12.5
General practice	5	12.5
Ophthalmology	4	10
Family medicine	3	7.5
Cardiology	3	7.5
Anesthesiology	2	5.0
Internal medicine	2	5.0
Abdominal surgery	1	2.5
Dermatology	1	2.5
General surgery	1	2.5
Gynecology	1	2.5
Oncology	1	2.5
Nuclear medicine and radiation protection	1	2.5
Physical and rehabilitation medicine	1	2.5
Public health	1	2.5
Radiology	1	2.5
Without specialization	1	2.5
